# Characterization of a Null Allelic Mutant of the Rice *NAL1* Gene Reveals Its Role in Regulating Cell Division

**DOI:** 10.1371/journal.pone.0118169

**Published:** 2015-02-06

**Authors:** Dan Jiang, Jingjing Fang, Lamei Lou, Jinfeng Zhao, Shoujiang Yuan, Liang Yin, Wei Sun, Lixiang Peng, Baotai Guo, Xueyong Li

**Affiliations:** 1 College of Life Sciences, Qingdao Agricultural University, Qingdao, China; 2 National Key Facility for Crop Gene Resources and Genetic Improvement, Institute of Crop Science, Chinese Academy of Agricultural Sciences, Beijing, China; 3 Shandong Rice Research Institute, Jinan, China; National Taiwan University, TAIWAN

## Abstract

Leaf morphology is closely associated with cell division. In rice, mutations in *Narrow leaf 1 (NAL1)* show narrow leaf phenotypes. Previous studies have shown that *NAL1* plays a role in regulating vein patterning and increasing grain yield in *indica* cultivars, but its role in leaf growth and development remains unknown. In this report, we characterized two allelic mutants of *NARROW LEAF1 (NAL1)*, *nal1-2* and *nal1-3*, both of which showed a 50% reduction in leaf width and length, as well as a dwarf culm. Longitudinal and transverse histological analyses of leaves and internodes revealed that cell division was suppressed in the anticlinal orientation but enhanced in the periclinal orientation in the mutants, while cell size remained unaltered. In addition to defects in cell proliferation, the mutants showed abnormal midrib in leaves. Map-based cloning revealed that *nal1-2* is a null allelic mutant of *NAL1* since both the whole promoter and a 404-bp fragment in the first exon of *NAL1* were deleted, and that a 6-bp fragment was deleted in the mutant *nal1-3*. We demonstrated that *NAL1* functions in the regulation of cell division as early as during leaf primordia initiation. The altered transcript level of G1- and S-phase-specific genes suggested that *NAL1* affects cell cycle regulation. Heterogenous expression of *NAL1* in fission yeast (*Schizosaccharomyces pombe*) further supported that *NAL1* affects cell division. These results suggest that *NAL1* controls leaf width and plant height through its effects on cell division.

## Introduction

To maximize the surface area directly exposed to light and enhance photosynthesis, leaves are typically flat and thin, and cell division has been studied for over a century as one of the decisive factors in leaf shape and size. Furthermore, molecular and genetic analyses have increased our understanding of the relationship between cell division and leaf morphology.

Leaf formation initiates from the shoot apical meristem (SAM; a small mound of undifferentiated tissue at the tip of the stem) and commences with the recruitment of founder cells in the peripheral zone at the flank of the meristem. This step is followed by cell wall expansion, periclinal cell division, and eventually induction of bulging at the leaf primordial [1–3]. P1 is the youngest leaf primordia, followed by P2, and so on in a developmental gradient. Leaf initiation is followed by the establishment of polarity, which includes proximodistal, adaxial–abaxial, and central–lateral axes [4]. Afterward, leaf primordia converts into mature leaves, which undergo two partially overlapping phases: cell expansion and division [5].

To date, many mutants with defects in cell division resulting in abnormal leaf shape and size have been characterized. In *Arabidopsis*, *ROTUNDIFOLIA4* (*ROT4*), which encodes a novel small peptide expressed on the plasma membrane, regulates cell proliferation along the longitudinal axis of the leaf. Either dominant mutations in *ROT4* or ectopic expression of *ROT4* result in short leaves [6]. Transcription factors play an important role in regulating cell division in leaves. GRF-INTERACTING FACTOR (GIF) proteins are putative transcriptional co-activators. In the small lateral organs of the *gif*1/2/3 triple mutant, the expression level of a B-type cyclin gene (*CYCB1;1*) and other cell cycle-related genes were significantly reduced, which decreased the number of cells in leaves, whereas *GIF1*, *GIF2*, and *GIF3* overexpression was responsible for the increased lateral organ growth of wild-type (WT) plants [7]. Assuming that the duration of cell proliferation in leaves is constant, altering the time to complete a single cell cycle should affect the total cell numbers in leaves, and therefore its final size [5]. The anaphase-promoting complex/cyclosome (APC/C) is known for its E3 ubiquitin-ligase activity, which regulates proteolysis of cell cycle regulators. Overexpression of a subunit of the APC/C complex, the APC10 protein, heightened APC/C activity, enhanced proteolysis of *CYCB1;1*, and augmented the rate of cell division in transgenic plants, which increased leaf size [8]. In addition, cell expansion resulting in larger cell size also played a role in leaf shape and size, in coordination with cell division. *GRF1* and *GRF2*, two members of the *GROWTH-REGULATING FACTOR* (*GRF*) family encoding putative transcription factors, interacted with *GIF*1 in *Arabidopsis*. The *grf1/2/3* triple loss-of-function mutant developed narrow and small leaves, whereas the overexpression of *GRF1* and *GRF2* resulted in larger leaves. The altered leaf shape and size was due to decreased and increased cell size, respectively [9].

The planes of cell divisions in plants can be divided mainly into anticlinal and periclinal orientations. Anticlinal cell divisions occur perpendicularly to the nearest surface, resulting in increased cell numbers, while periclinal cell divisions occur parallel to the nearest surface and lead to escalated cell layers. The plane of cell division for most plant cells depends largely on the positions in which new cell walls are formed [10, 11]. In barley (a monocot), the *elongation* (*elo*) mutant seedlings did not respond to gibberellic acid (GA_3_) at any concentration. Leaves of the mutant *elo2* and *elo-5* contained reduced cellulose in leaf cell walls and exhibited aberrant periclinal cell divisions, which resulted in the formation of increased cell layers in the leaf epidermis. Misplaced anticlinal divisions also were observed in the *elo-5* leaf epidermis [12, 13]. Also in monocots, *Extra cell layers1* (*Xcl1*) is a semidominant mutation in maize. Overproduction of the *XCL1* gene product resulted in aberrant oblique periclinal divisions in the protoderm layer. This promoted protodermal periclinal divisions was accompanied by inhibited normal anticlinal divisions and resulted in extra cell layers with epidermal characteristics [14]. A recent study showed that ectopic expression of the auxin-regulated basic helix–loop–helix (bHLH) transcription factor is sufficient to trigger periclinal divisions in vascular tissue, whereas bHLH-defective mutants show a loss of periclinal divisions [15].

In rice, *NARROW LEAF 1* (*NAL1*), which encodes a putative trypsin-like serine and cysteine protease [16], affects vein patterning and polar auxin transport (PAT) [17]. Previous studies have shown some major quantitative trait loci (QTL) are allelic to *NAL1*. Chen reported that the major QTL *FLAG LEAF WIDTH* (*qFLW*) is allelic to *NAL1*. The functional difference between the two alleles was due to alternative splicing [18]. The QTL *GREEN FOR PHOTOSYNTHESIS* (*GPS*) in rice, which is allelic to *NAL1*, was shown to control photosynthesis by regulating carboxylation efficiency [19]. Similarly, more recent reports have shown that QTLs *SPIKELET NUMBER* (*SPIKE*) and *LSCHL4* were both allelic to *NAL1*. An near-isogenic line carrying *SPIKE* or *LSCHL*4 in the *indica* background (NIL-*SPIKE* or NIL*-LSCHL4*) achieved yield increase through improving yield-related traits [20, 21]. Thus, the *NAL1* gene could be applicable for rice breeding.

Here, we present a detailed analysis of cellular characteristics underlying the narrwow leaf and semi-dwarf phenotype in the rice mutant *nal1-2* and *nal1-3*. We found that mutations in *NAL1* disturb both anticlinal and periclinal cell divisions in leaves and internodes and that *NAL1* plays a role in cell division as early as during leaf primordia initiation.

## Materials and Methods

### Plant materials and growth conditions

The mutants were screened from the *japonica* cultivar Nipponbare, seeds of which was treated with gamma ray irradiation with a dose of 200 Gy and dose rate of 10 Gy. h^-1^. The mutant plants were sowed in the Experimental Field of the Shandong Rice Research Institute (Jining, Shandong, China) and grown under normal field management.

### Epidermal cell observations

We treated materials as described by [22]. Approximately 2 months after sowing, the upper second leaf blades of *nal1-2* and WT plants were fixed in formaldehyde:glacial acetic acid:50% ethanol (FAA; 2:1:17) solutions overnight, followed by dehydration in a graded ethanol series. The dehydrated leaf blades were then incubated at 96°C in chloral hydrate dissolved in 100% ethanol. We then scraped the leaf blades with a double-edge blade until the abaxial epidermis was exposed. The specimens were then cropped and transferred to glass slides with small drops of water and examined under a light microscope.

### Histological analysis of the shoot apex

The 4-day-old seedlings (grown on ½ Murashige and Skoog medium) were fixed in the FAA solution overnight at 4°C, dehydrated in a gradient series of ethanol, and cleared through a xylene series. The samples were finally embedded in 100% Paraplast (Sigma, St. Louis, MO, USA) at 55–60°C. Microtome sections (7–9 μm thickness) were mounted to poly-L-coated slides (Sigma), dewaxed in xylene, rehydrated through an ethanol series, stained with 1% Fast Green, and finally stored in Canada balsam for light microscopic examination.

### Investigation of internodes and leaves

During the heading stage, internodes and leaves were collected from plants in the field and briefly conserved in water to prevent moisture loss. We adopted the free hand-sectioning method to conduct all transverse and longitudinal sections of internodes and leaves. The materials were processed in the fresh state immediately after collection. We sectioned the target part of samples, chose several ideal sections from each sliced individual sample, and then observed and photographed them under a light microscope.

### Statistics of size and number of cells

A system microscope (BX43; Olympus, Tokyo, Japan) and cellSens Standard software (Olympus) allowed us to observe and measure cells and tissues. Sections of samples were observed under the BX43 and then photographed under the proper objective. Using measurement functions of cellSens Standard software, we measured the size and number of cells.

### Map-based cloning

The candidate gene was mapped primarily using 20 F_2_ mutant plants. Fine mapping was performed with 1948 F_2_ individuals. We developed nine molecular markers based on the sequence difference between *japonica* variety Nipponbare (http://rgp.dna.affrc.go.jp/) and *indica* variety Dular (unpublished data). The primer sequences of the molecular markers are listed in S1 Table. We applied a Genome Walking Kit (TaKaRa) to conduct genome-walking trials. Specific primers (SPs) (located 200 bp downstream of the first exon of *NAL1* ORF) were designed as described by the manufacturer. We then performed three steps of PCRs following the manufacturer’s instructions. SP primer sequences are listed in S1 Table. The DNA products from final step PCR were cloned into a pMD19-T Vector (TaKaRa) for sequencing.

### Complementation test

The full-length coding sequence of *NAL1* was amplified using primers *NAL1*-F: (5′-CGAACGATAGCCATGGGCATGAAGCCTTCGGACGATAAG-3′) and *NAL1*-R (5′-GGGTAGGATCCACTAGTTTTCTCCAGGTCAAGGCTTG-3′), and the PCR fragment was inserted downstream of the rice *ACTIN 1* promoter in the vector pCAMBIA1305.1AP. The resulting plant expression vector was introduced into the *nal1-2* mutant via the *Agrobacterium tumefaciens—mediated transformation method*.

### Quantitative real-time PCR (qPCR) analysis

Total RNA was isolated using TRIzol solution (Invitrogen, Carlsbad, CA, USA) from the leaves of 10-day-old seedlings of the WT and the *nal1-2* plants. First-strand cDNA was synthesized from 2 μg of total RNA primed with the oligo-dT primer using the Superscript III Reverse Transcription Kit (Invitrogen). Semiquantitative RT-PCR was performed with LA Taq DNA polymerase (TaKaRa) using the rice *Ubiquitin* gene (*LOC_Os*02g*06640*) as an internal control.

qPCR analysis was performed with a SYBR Premix Ex Taq2 (TaKaRa) kit and run on ABI PRISM 7900HT (Applied Biosystems, Foster City, CA, USA). The transcript levels of the examined genes were normalized to the rice *Ubiquitin* gene and the relative expression levels were compared with WT plants. All primers used in qPCR analyses are listed in S1 Table.

### Heterogenous expression of *NAL*1 in fission yeast

The full-length coding sequence of *NAL1* was amplified using primer NAL(PREP)F (5′-TTAAATCATATGTCGACATGAAGCCTTCGGACGATAAG-3′) and NAL(PREP)R (5′-CTTTTACCCGGGGATCCTCATTTCTCCAGGTCAAG-3′). The PCR product was cloned into the yeast expression vector pREP1 [23] and verified by sequencing. Expression of *NAL*1 in *S*. *pombe* was induced by the absence of thiamine as reported [24].

## Results

We identified two allelic mutants of *NAL1* designated as *nal1-2* and as *nal1-3*, respectively. We mainly focused on the analysis of the *nal1-2* mutant. The *nal1-3* mutant has similar phenotype to *nal1-2* and the data were briefly shown in S5 Fig. and in S2 Table.

### The mutant nal1-2 showed severe phenotypes

**Fig 1:**
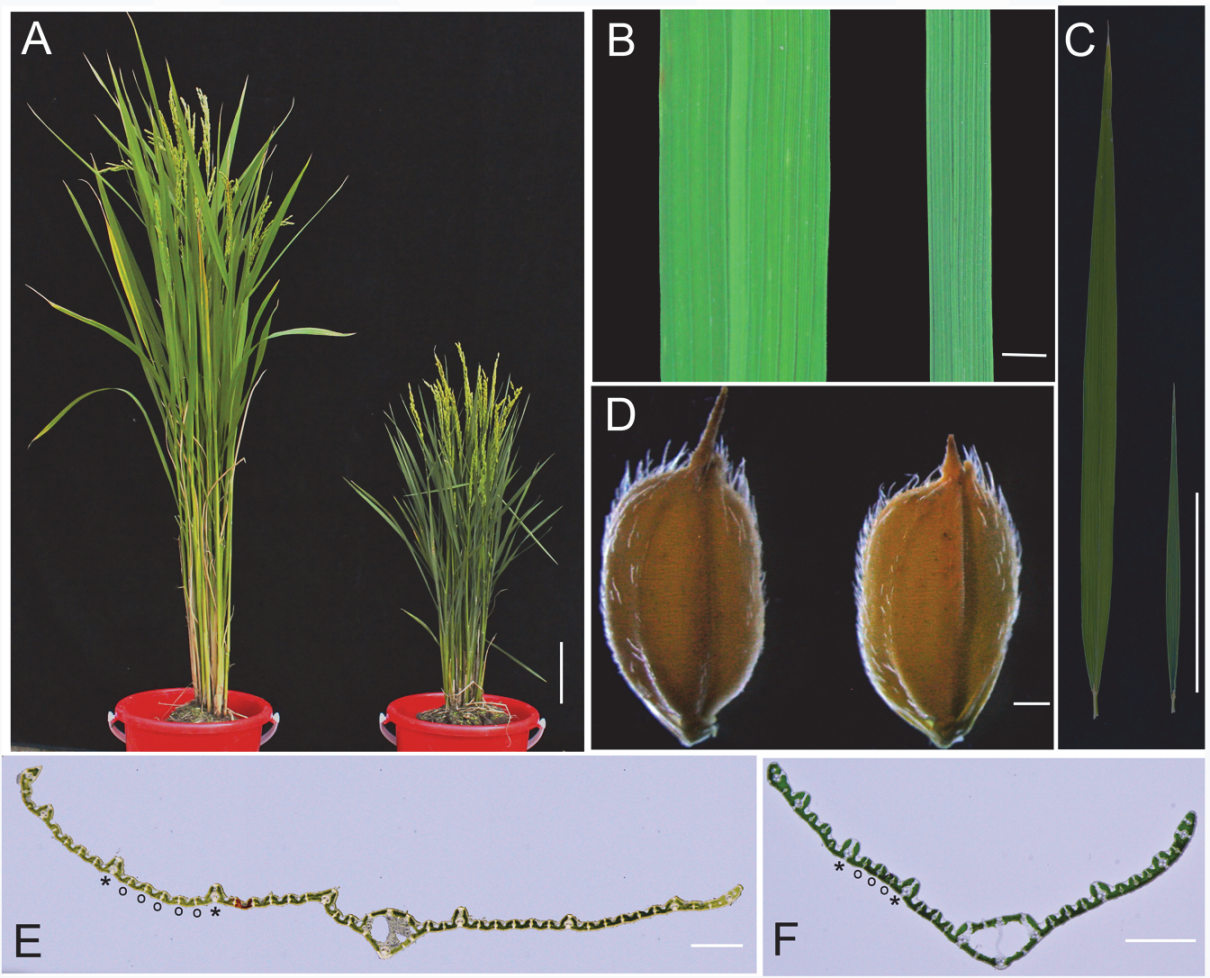
Phenotypes of *nal1-2*. (A) Morphology of wild-type (WT; Nipponbare, left) and *nal1-2* (right) plants at the heading stage (bar = 5 cm). (B) Comparison of leaf width between the WT (left) and *nal1-2* (right) (bar = 5 mm). (C) Comparison of leaf length between the WT (left) and *nal1-2* (right) (bar = 5 cm). (D) Comparison of seed phenotype between the WT (left) and *nal1-2* (right) (bar = 0.5 mm). (E, F) Transverse sections through the middle part of the mature upper second leaves of WT (E) and *nal1-2* plants (F) (bars = 1 mm). Asterisk and circle in E and F indicate large vein and small vein, respectively.

The phenotypes of *nal1-2* mainly showed reduced leaf width and length by more than 50% compared with the WT (Fig. 1A, B, C; Table 1). The leaf index (the ratio of the leaf length to width) of flag leaves in *nal1-2* was normal, which suggested that the shape of leaves in *nal1-2* was not affected, similar to seed shape and size (Fig. 1C, D; Table 1). The amount of large and small veins within the upper second leaf in *nal1-2* reached only 68% and 32% of their WT counterparts, respectively (Fig. 1E, F; Table 1). Tiller number in *nal1-2* approached approximately 240% compared to the WT at the filling stage (Table 1). The decrease in plant height in *nal1-2* was evident. As shown in Fig. 1A and Table 1, the height of *nal1-2* was reduced by approximately 50% compared to the WT. These results indicated that the mutant *nal1-2* possesses severe phenotypes.

**Table 1:** Morphometric analysis of the ***nal1-2*** mutant.

Traits	Wild Type	nal1-2
Plant height (cm)	105.73±1.84	56.76±1.97**
Blade length of flag leaf (cm)	37.24±2.50	15.67±1.27**
Blade length of second leaf (cm)	46.24±1.78	26.50±1.17**
Blade width of flag leaf (mm)	15.31±0.61	6.86±0.36**
Blade width of second leaf (mm)	14.85±0.68	5.64±0.36**
Tiller number	13.42±1.75	31.60±3.23**
Number of large veins in second leaf	11.40±1.13	7.78±0.76**
Number of small veins in second leaf	46.9±2.65	15.23±0.82**
Leaf index of flag leaf	24.33±1.57	22.82±2.03

Phenotypes of *nal1-2* were measured at filling stage. Seconde leaf is the upper second leaf. Values are the mean ± standard error (SE) (n≥15). Asterisks reveal the significance of differences between wild-type and *nal1-2* plants, which is obtained by Student’s *t*-test: *, 0.01 ≤ *P* < 0.01; **, *P* < 0.01.

### Periclinal and anticlinal cell division in leaves in nal1-2

**Fig 2:**
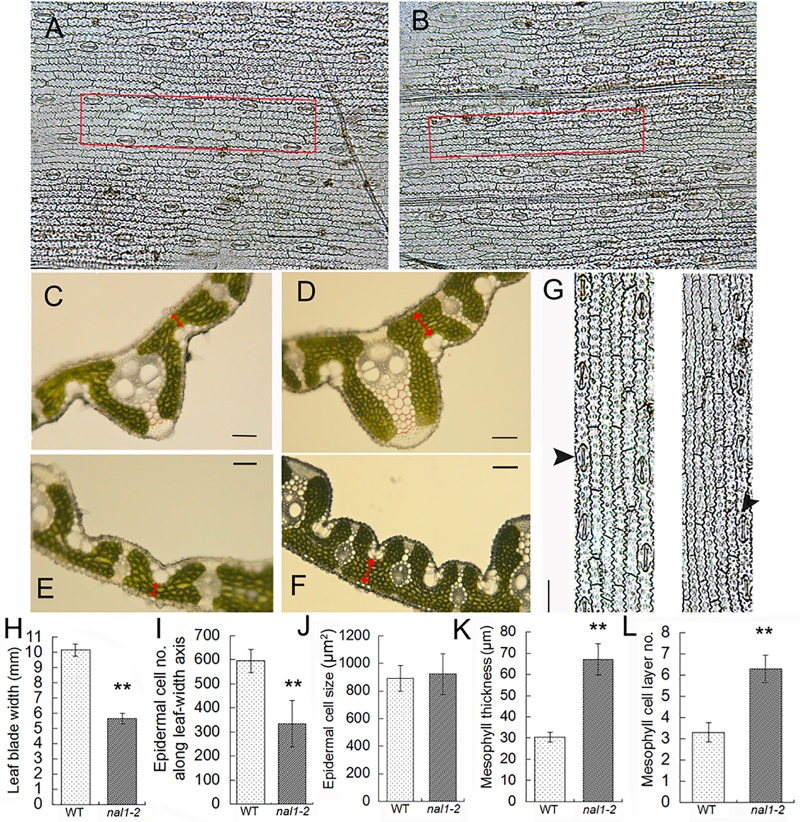
Histological analyses of leaves. (A, B) The abaxial epidermis of the upper second leaf blade of wild-type (WT) (A) and *nal1-2* (B) plants (bars = 50 μm). (C–F) Cross sections through the middle part of the upper second leaf blade of WT (C, E) and *nal1-2* (D, F) plants. Red double-headed arrows indicate the thickness of the leaf mesophyll adjacent the midrib (C, D) and small veins (E, F). Red curves in C and D outline the parenchyma cell layers (bars = 50 μm). (G) Magnified images of the red-boxed regions in A (left) and B (right). Black arrows show the position of stomatal guard cells (bar = 50 μm). (H–L) Comparison of the leaf blade width (H), epidermal cell number (I), epidermal cell size (J), leaf mesophyll thickness (K), and the number of mesophyll cell layers (L) between the WT and *nal1-2*. Epidermal cell number was calculated by dividing each leaf blade width by the corresponding epidermal cell width. Data are shown as means ± standard error (SE) (H, I, K, and L, n ≥ 10; J, n ≥ 500). Student’s t-test was used to analyze significant differences between the WT and *nal1-2*. *, 0.01 ≤ P < 0.01; **, P < 0.01.

In rice, the leaf is composed of different cell layers, including the epidermis, mesophyll, and vascular tissues [25]. Anticlinal–transverse cell divisions broaden leaf width, whereas anticlinal–longitudinal cell divisions elongate leaf length. Epidermis differentiated from the outermost layer (L1) of the SAM consists of a single cell layer, which exhibits mostly anticlinal cell divisions [26, 27]. We examined the number of abaxial–epidermal cells at the middle part of the upper second leaves in the anticlinal–transverse orientation (along leaf-width axis) in the mutant *nal1-2* (Fig. 2A, B). Compared with the WT, the epidermal cell size, cell length, and cell width were unchanged in the mutant *nal1-2* (Fig. 2G, J; S1A-B Fig.). In addition, no observable difference was detected between stomatal guard cells (Fig. 2G). Calculating the leaf blade width and cell width allowed us to estimate the number of cells within the leaf blade in the anticlinal–transverse orientation [28]. As shown in Fig. 2H-I, the quantity of abaxial–epidermal cells in *nal1-2* had decreased by approximately 44% compared to WT leaves. Therefore, the diminished width of the leaf blade in the *nal1-2* was caused by defects in anticlinal cell divisions.

In addition to these observations of anticlinal cell divisions in *nal1-2*, we examined cell divisions in the periclinal orientation. Previous reports showed that *Nal1* affects mesophyll cells in a manner that alters cell quantity [19]. Based on a series of images of cross sections, we noted that the number of mesophyll cell layers within the *nal1-2* increased significantly compared to the WT (Fig. 2C–F). The number of parenchyma cell layers within large veins also increased in *nal1-2* (Fig. 2C, D). We manually counted the number of mesophyll cell layers and measured the thickness of mesophyll tissue. The number of mesophyll cell layers within leaves of the *nal1-2* was significantly higher than the WT counterparts (Fig. 2L). We observed three mesophyll cell layers between two small veins in most WT leaves, whereas the mutant leaves had six layers. As a result, the thickness of mesophyll tissue, which is composed of mesophyll cells, in the mutant *nal1-2* was 120% greater than in the WT (Fig. 2K). The number of mesophyll cell layers was also increased in the mutant *nal1-3* (S5F Fig.). Therefore, the *nal1* mutants showed an enhanced cell proliferation in the periclinal direction and suppressed cell division in the anticlinal direction.

### NAL1 plays a role in leaf development as early as during leaf primordia initiation

**Fig 3:**
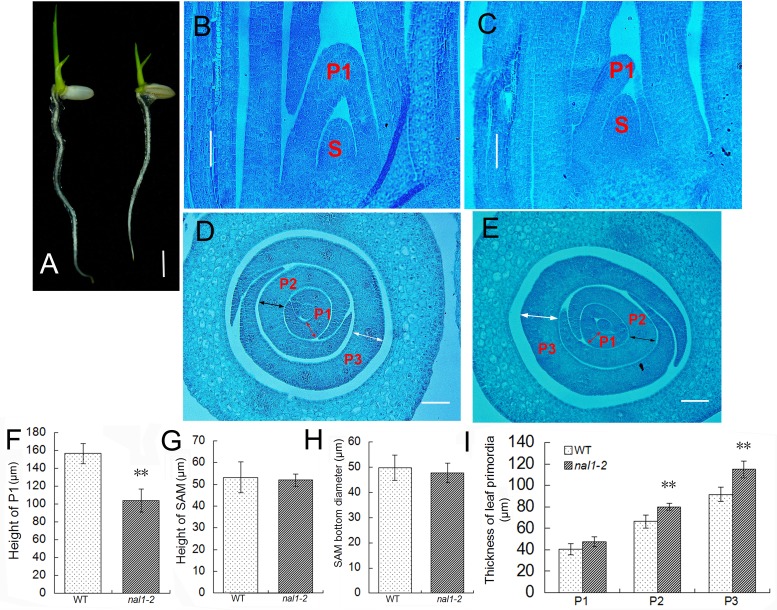
Repressed leaf primordia growth in the *nal1-2*. (A) The phenotypes of 6-day-old seedlings from the wild type (WT; left) and *nal1-2* (right) (bar = 5 mm). (B, C) Longitudinal sections of shoot apexes of WT (B) and *nal1-2* (C) plants (bars = 50 μm). (D, E) Transverse sections of shoot apexes of WT (D) and *nal1-2* (E) plants. Red, black, and white double-headed arrows indicate the thickness of P1, P2, and P3, respectively (bars = 50 μm). S, P1, P2 and P3 represent the shoot apical meristem (SAM) and P1, P2, P3 leaf primordia, respectively. (F–I) Comparison of the height of P1 between the WT and *nal1-2* (F), and SAM (G), diameter of the bottom of the SAM (H), and the thickness of leaf primordia (I). Each column represents the mean ± standard error (SE) (F–H, n ≥ 10; I, n ≥ 5). Asterisks show significant differences between WT and *nal1-2* plants based on Student’s t-test: *, 0.01 ≤ P < 0.01; **, P < 0.01.

Although we found that *NAL1* antagonistically regulates periclinal and anticlinal cell divisions during leaf growth, the function of *NAL1* during leaf primordia development remained unclear. We found that *nal1-2* showed dwarf phenotypes as early as the seedling stage (Fig. 3A). Thus, 4-day-old seedlings were collected and fixed in FAA to obtain both cross and longitudinal sections. In a series image of longitudinal sections through the shoot apexes, we detected no difference (in shape or size) in the SAM between the WT and *nal1-2* (Fig. 3B, C, G, H). However, we noted that the height of P1 leaf primordia in *nal1-2* was shorter than in the WT (Fig. 3B, C). We measured the height of P1 leaf primordia in more than 10 seedlings and found that the average height of P1 in *nal1-2* reached approximately 66% that of the WT, suggesting that the suppressed cell division in the anticlinal orientation in the *nal1-2* had already affected the development of P1 leaf primordia (Fig. 3F). Observations of the transverse sections of the shoot apexes corroborated this result (Fig. 3D, E). The thickness of the middle part of the P1, P2, and P3 leaf primordia in the transverse direction were measured. Compared to the WT, the thickness of P1 leaf primordia increased slightly in *nal1-2*, while P2 and P3 leaf primordia in the mutant approached approximately 121% and 125%, respectively, compared to their WT counterparts (Fig. 3I). These results suggest that the *NAL1* gene plays a role in leaf development as early as during leaf primordia initiation, but the structure of the SAM is not affected by *NAL1*.

### Cell division in culms

**Fig 4:**
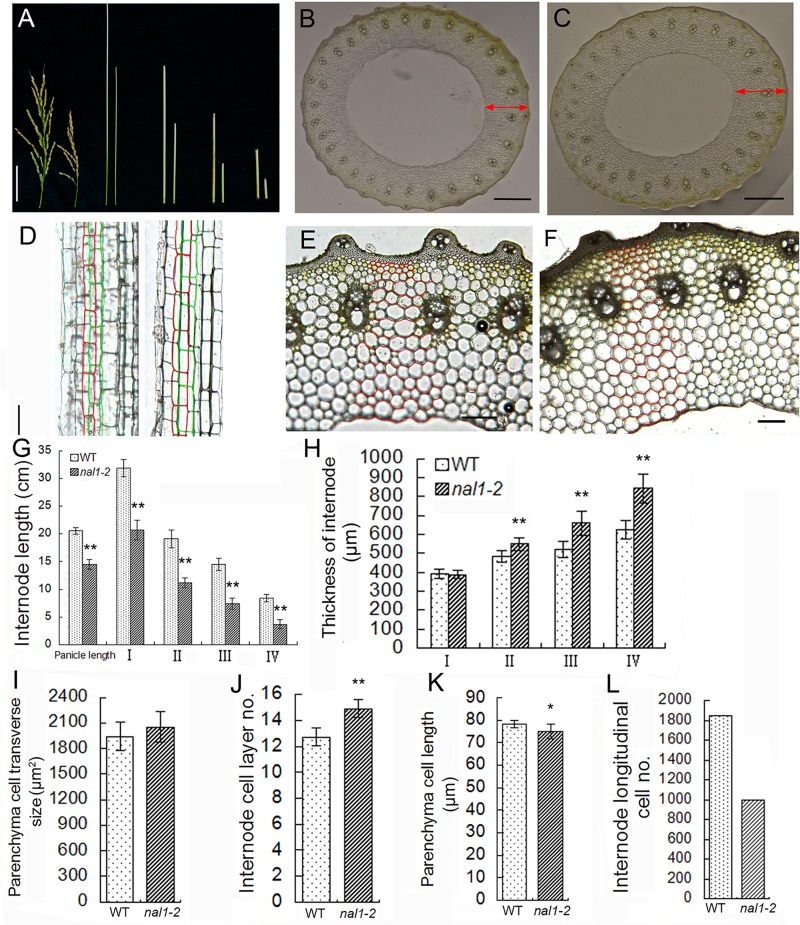
Proliferative status of cell divisions in the internode of *nal1-2*. (A) The lengths of the panicle and internodes are compared between the wild type (WT; left) and *nal1-2* (right; bar = 5 cm). (B, C) Cross sections through the middle part of the internode III in the WT (B) and *nal1-2* (C). Red double-headed arrows indicate the thickness of the internode III. Bars = 0.5 mm. (D) Comparison of longitudinal sections of internode III between the WT (left) and *nal1-2* (right). Red and green boxes outline parenchyma cells (bar = 50 μm). (E, F) Images of transverse sections of internode are zoomed-in. Cell layers of the WT (E) and *nal1-2* (F) are contoured by red curves (bars = 100 μm). (G–L) Comparison of internodes length (G), internodes thickness (H), parenchyma cell size along the transverse axis (I), the number of internode III cell layers (J), and parenchyma cell length along the longitudinal axis (K) in internode III between the WT and *nal1-2*. I–IV represents the upper four internodes, respectively. Results are shown as means ± standard error (SE) (G–J, n = 10; I, n ≥ 230; K, n ≥ 200). Asterisks represent significant differences between WT and *nal1-2* plants based on Student’s t-test: *, 0.01 ≤ P < 0.01; **, P < 0.01. (L) The number of cells along the longitudinal axis in internode III. Data were estimated by dividing the average internode length by the average parenchyma cell length.

As shown in Fig. 1A, a dwarf culm was observed in *nal1-2*. The length of panicle and internodes I, II, III, and IV in *nal1-2* were shortened by 29%, 35%, 41%, 49%, and 56%, respectively (Fig. 4A, G). We conducted a transverse histological investigation on the upper four internodes and longitudinal histological investigation on internode III at the filling stage. Although the average length of parenchyma cells was slightly shortened in *nal1-2* (Fig. 4K), the number of cells along the longitudinal axis reached approximately 53% compared to the WT (Fig. 4D, L). In transverse sections of the internode, the distribution pattern of vascular bundles in the mutant *nal1-2* was normal, as well as the structure of the vascular bundle (Fig. 4B, C). Similar to leaves, thickness of the internode increased in the mutant *nal1-2*, as well as in the mutant *nal1-3* (Fig. 4B, C; S5D Fig.). Since the form of transverse sections of the internode was similar to a circle, we measured both the outer and inner diameters of the upper four internodes (S1C-D Fig.). Using the outer diameter and inner diameter of internodes, we calculated the internode thickness. The thicknesses of internodes II, III, and IV (excluding internode I) in *nal1-2* increased compared to the WT. Additionally, these rates soared from 13.1% in internode II to 35% in internode IV (Fig. 4H). The difference in cell size between *nal1-2* and the WT was almost undetectable (Fig. 4I). We manually recorded the number of cell layers in internode III (Fig. 4E, F). The average number of cell layers in internode III in *nal1-2* was significantly greater than in the WT (Fig. 4J). These results indicated that the decrease in cell numbers in the anticlinal orientation is the primary reason for shortened internodes in *nal1-2*. The increase in the number of cell layers in the periclinal orientation resulted in thickened internodes in *nal1-2*. Therefore, *nal1* mutation enhanced cell proliferation in the periclinal direction and suppressed cell division in the anticlinal direction in the internode as well as in leaf, but was not involved in the regulation of cell expansion.

### Effects of NAL1 on the formation of normal leaf midribs

**Fig 5:**
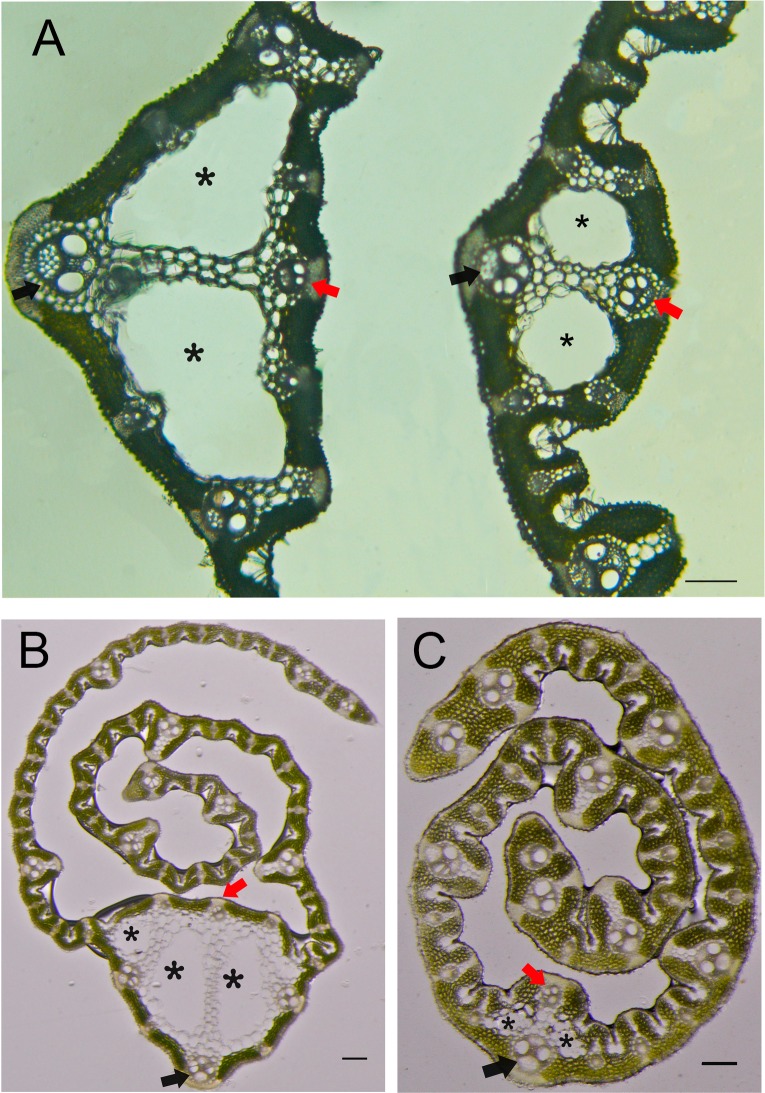
Formation of an abnormal leaf midrib in the *nal1-2*. (A) Cross sections through the middle part of mature leaves in the WT (left) and *nal1-2* (right). (B, C) Transverse sections through the upper part of young flag leaves in wild-type (WT) (B) and *nal1-2* (C) plants. Black asterisks indicate clear cells, red arrows show adaxial small vascular bundles, and black arrows denote the central large vascular bundle (bars = 0.1 mm).

The midrib structure in rice leaves possess two characteristics. First, two large locules (called clear cells) are in the central region of the leaf midrib, which are formed by programmed cell death. Second, the vascular bundle on the adaxial side is notably smaller than the one on the abaxial side (Fig. 5A) [29, 30]. Within mature flag leaves in the mutant *nal1-2*, the area consisting of clear cells was significantly smaller than that in the WT. In addition, the size of the vascular bundle on the adaxial side in the *nal1-2* was similar to the opposite side (Fig. 5A). Moreover, these abnormal phenotypes were more evident in young leaves (Fig. 5B, C). The same number of large veins manifested at the same stage of growth. As the clear cells in the WT young leaf (containing nine large veins) had almost completed programmed cell death, the pattern of the midrib was the same as in mature leaves. However, this process was postponed in the midrib of the mutant *nal1-2* leaf (which also contained nine large veins). The formation of clear cells was incomplete at this stage, which significantly reduced the cell size. Meanwhile, the status of vascular bundles on both the adaxial and abaxial sides was similar to mature leaves in *nal1-2*. These observations indicated that *NAL1* regulates vascular bundle formation of a normal leaf midrib.

### nal1-2 was a null allelic mutant of the Narrow Leaf 1 gene

**Fig 6:**
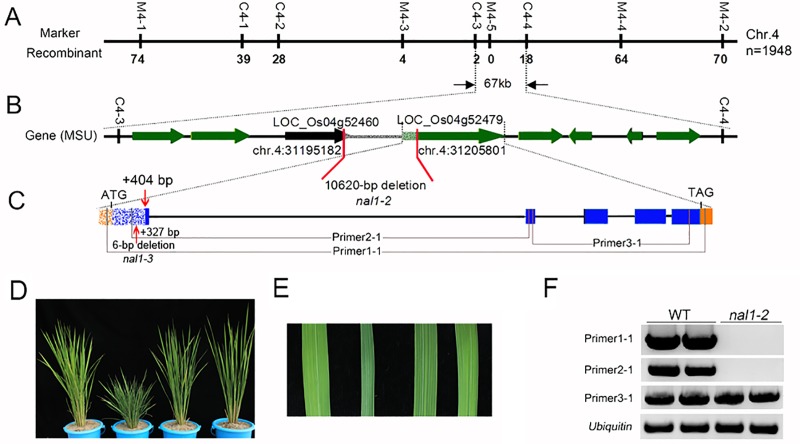
Map-based cloning of *nal1-2*. (A) The gene responsible for the *nal1-2* phenotype was located in an approximately 67-kb region on chromosome 4 (chr. 4). Vertical lines indicate the positions of molecular markers and the number of recombinants. (B) Seven putatively functional open reading frames (ORFs) and a retrotransposon within the fine mapping region. Red broken lines represent the position of the deleted fragment. The deleted region is modified by fragmentation. Green arrows represent putatively functional genes and the black arrow points to the retrotransposon. (C) Both the whole promoter and part of the first exon of LOC_Os04g52479 (*NAL1*) were deleted in the *nal1-2* mutant. A 6-bp deletion located in the first exon was also detected in the *nal1-3* mutant. The letters ATG and TAG represent the start and stop codon, respectively. The red arrow shows the edge of the deleted fragment. Yellow and blue boxes represent the regulatory region and ORF, respectively. Gray lines indicate the positions of primers designed for semiquantitative RT-PCR analyses. (D, E) Genetic complementation of *nal1-2*. From left to right are WT, *nal1-2*, and two representative lines of transgenic plant. (D) Gross morphology at the early heading stage showing complementation of plant height. (E) Central part of flag leaves showing complementation of leaf width. (F) The transcript level of *NAL1* was detected by semi-quantitative RT-PCR analyses. Total RNAs were isolated from the first two leaves of WT and *nal1-2* plants. Ubiquitin was used as an amplification control.

Genetic assays to determine the segregation ratio of F_2_ progenies obtained from a cross between the mutant *nal1-2* and dular (an *indica* rice cultivar) showed that *nal1-2* possesses a recessive mutation in a single locus. Primary mapping using 20 F_2_ homozygous plants located the *nal1-2* locus on the long arm of chromosome 4 between markers RM-30.1 and RM-32.6 (data not shown). Fine mapping of *nal1-2* was performed with 1948 F_2_ individuals showing mutant phenotypes. We developed nine molecular markers (S1 Table) to narrow the *nal1-2* locus within a 67-kb region (Fig. 6A). Within this 67-kb interval, according to the MSU Rice Genome Annotation Project (http://rice.plantbiology. msu.edu/), are a retrotransposon and seven putative expressed genes, including *Narrow Leaf 1* (LOC_Os04g52479) (Fig. 6B) [17]. We targeted LOC_Os04g52479 as a candidate gene for the mutant *nal1-2* and sequenced its DNA. However, we could not obtain the fragment containing the first exon of the candidate gene through ordinary polymerase chain reaction (PCR). We hypothesized that the first exon of LOC_Os04g52479 ORF was completely or partially deleted during radiant inducement. The Genome Walking Kit (TaKaRa, Kyoto, Japan) was used to validate our hypothesis. We sequenced several DNA fragments obtained through genome-walking trials (S2 Fig.). Blasting these results in the MSU Rice Genome Annotation Project (http://rice.plantbiology.msu.edu/) revealed that the *nal1-2* mutant was lacking a 10,620-bp fragment (from chr.4:31195182 to chr.4:31205801), within which the whole promoter and 404-bp fragment of the first exon of *NAL1* were contained (Fig. 6B, C). To confirm this result, we designed a pair of primers (primer4-1) (S1 Table). The forward primer4-1 was complementary to the sequence upstream of chr.4:31195182, whereas the reverse primer4-1 was complementary to the sequence downstream of chr.4:31205801. We obtained the predicted size of the DNA fragment in *nal1-2* using PCR with primer4-1 (S3 Fig.). In another mutant *nal1-3*, a 6-bp deletion (cgtttt) in the first exon of the *NAL1* gene was also detected, resulting in the missing of two amino acids (R110 and F111) (Fig. 6C). Alignment of NAL1 with homologous proteins indicated that the deleted sequences are highly conserved among angiosperms (S4 Fig.).

To verify whether the deletion in LOC_Os04g52479 is responsible for the mutant phenotype, we carried out a complementation test by introducing the wild-type *NAL1* coding sequence driven by the rice *ACTIN 1* promoter into the *nal1-2* mutant. Nine out of ten transgenic lines showed complementation of the *nal1-2* phenotypes (Fig. 6D, E), confirming that *NAL1* is indeed the candidate gene.

RT-PCR was performed to determine alteration of the *NAL1*transcript in the *nal1-2* mutant. Three pairs of primer were designed, primer 1-1 to amplify full length cDNA, primer 2-1 to amplify cDNA sequence from the first to the second exon, and primer 3-1 to amplify cDNA sequence from the second to the last exon. Although the 3’ fragment can be transcribed (pirmer 3-1), no transcript from the 5’ fragment (primer 2-1) or full length coding sequence (primer 1-1) could be detected (Fig. 6F). Thus, the *NAL1* transcript in *nal1-2* was incomplete and *nal1-2* is a null allele.

### Altered expression level of cell division- and auxin-related genes in the nal1-2 mutant

**Fig 7:**
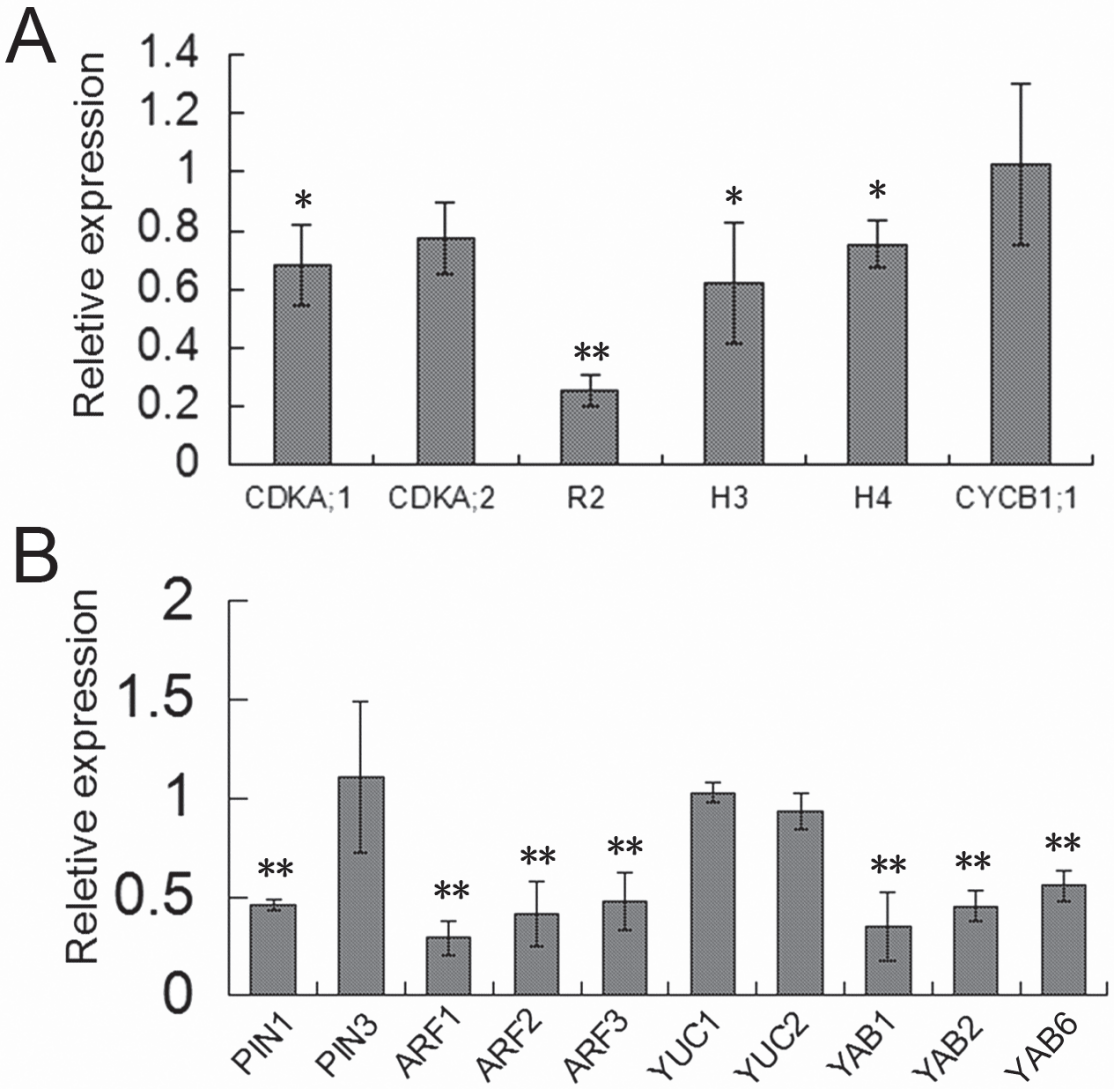
Altered expression of genes related to cell division and abaxial–adaxial polarity in *nal1-2*. The rice *Ubiquitin* gene was used as an internal positive control and the expression level of each gene was normalized to *Ubiquitin*. (A) Quantitative PCR (qPCR) analysis was performed to study the transcript levels of cell division related genes in leaves of wild-type (WT) and *nal1-2* plants. Total RNAs were isolated from leaves of 10-day-old seedlings. (B) qPCR analysis of the transcripts of auxin-related genes (*PIN* family, *ARF* family, and *YUCCA* family) and abaxial–adaxial polarity-related genes (*YABBY* family) in leaves of of 10-day-old seedlings of WT and *nal1-2* plants. Each column represents the mean ± standard error (SE) of three independent assays. Asterisks show significant differences between WT and *nal1-2* plants based on Student’s *t*-test: *, 0.01 ≤ *P* < 0.05; **, *P* < 0.01.

The altered rate of cell proliferation in the *nal1-2* mutant prompted us to examine the expression of cell cycle-regulating genes. Total RNA was isolated from the leaves of 10-day-old seedlings of WT and *nal1-2* plants, and was subjected to RT-PCR. Expression level of the Cyclin-dependent kinase-activating kinase *R2* which regulates S-phase progression [31] was decreased by nearly two-thirds in *nal1-2* compared to the WT (Fig. 7A). Similarly, expression level of two S-phase specific marker genes *H3* and *H4* [32] and two marker genes of the G1- to S-phase transition *CDKA;1* and *CDKA;2* [33, 34] were also decreased in *nal1-2* (Fig. 7A). However, no difference was observed in the expression level of *CYCB1;1*, the marker gene of the G2- to M-phase transition [35–37] between the WT and mutant *nal1-2* (Fig. 7A). Therefore, these results indicated that the altered rate of cell proliferation in *nal1-2* might be due to the altered expression level of G1- and S-phase-specific genes.

Previous studies have shown that *NAL1* plays a role in the regulation of PAT and affects the expression level of *PIN1* (a well-characterized auxin efflux carrier) [17, 38]. Thus, we investigated expression of other auxin-related gene in *nal1-2*. Similar to *nal1*, the expression level of *PIN*1 in the *nal1-2* mutant was reduced to half compared to the WT (Fig. 7B). However, the expression level of *PIN3* (another auxin efflux carrier), as well as *YUCCA1* and *YUCCA2* (which catalyze a rate-limiting reaction in tryptophan-dependent auxin biosynthesis), was not significantly affected (Fig. 7B) [39, 40]. However, the expression levels of three members of the *AUXIN RESPONSE FACTOR* (*ARF*) gene family (*ARF1*, *ARF2*, and *ARF3*) and three members of the *YABBY* gene family (*YABBY1*, *YABBY2*, and *YABBY6*) (involved in establishing abaxial–adaxial polarity in lateral organs) in *nal1-2* were reduced by almost 50% compared to the WT (Fig. 7B) [4, 41, 42]. Thus, *NAL1* appears to play a role in both polar auxin transport and auxin response, but did not regulate the tryptophan-dependent auxin biosynthesis. Previous studies have shown that a member of the *YABBY* gene family, *DROOPING LEAF* (*DL*), is required for midrib formation in leaves. Overexpression of *DL* led to ectopically formed clear cells, as well as abnormal adaxial vascular bundles [43]. Thus, the formation of an abnormal midrib in *nal1-2* leaves may be correlated with the downregulation of *YABBY* expression.

### NAL1 involves in cell cycle regulation

**Fig 8:**
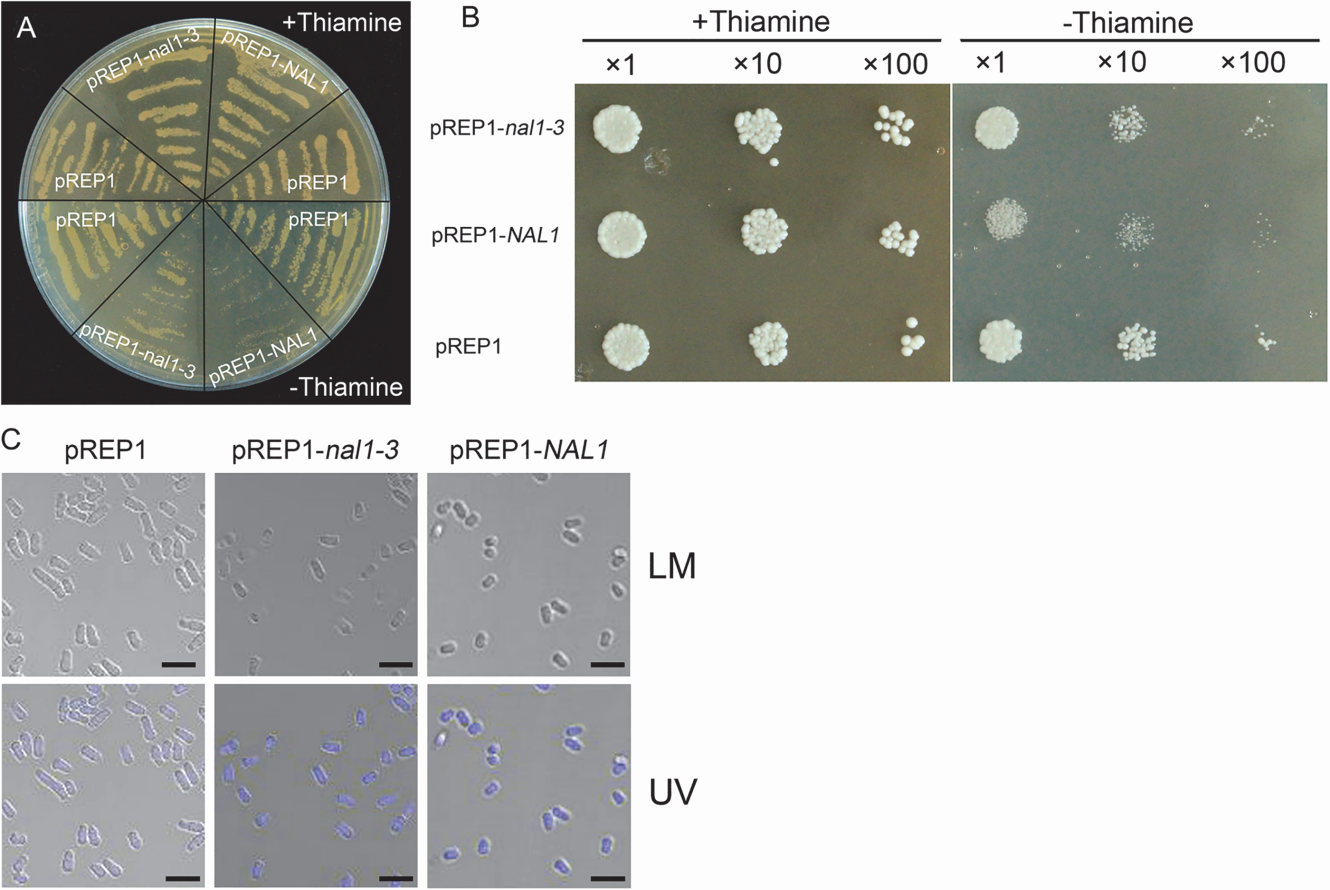
Heterologous expression of *NAL*1 in yeast (*S*. *Pombe*). (A, B) Growth of *S*. *pombe* transformed with *pREP1-NAL1*, *pREP1-nal1-3*, and the empty vector *pREP*1 in the presence (+thiamine, the expression of gene is repressed) and absence of thiamine (-thiamine, the expression of gene is induced). (A) Spreading yeast cells on the plate. (B) Spotting yeast cells on the plate after a serial of dilution. (C) Phenotype of yeast cells under bright and UV light microscopy in the absence of thiamine. LM, light microscopy; UV, observation of 4’,6-diamidino-2-phenylindole (DAPI) stained cells under UV light (bars = 10 μm).

To investigate the possible effect of *NAL1* on cell cycle regualtion, we heterogenously expressed *NAL1* gene in fission yeast (*Schizosaccharomyces pombe*), an excellent model system for analysis of mechanisms of cell cycle regulation. The *NAL1* complementary DNA (cDNA) was cloned into the yeast expression vector pREP1 and overexpressed in the absence of thiamine. As shown in Fig. 8A, B, overexpression of *NAL*1 in fission yeast resulted in severe growth arrest. Microscopic observation showed that yeast cell number was clearly reduced while cell size remained almost unaltered (Fig. 8C), indicating that the process of cell division in fission yeast is inhibited. However, overexpression of the mutant allele *nal1-3* which harbors two amino acids deletion exerted milder inhibitory effect on the yeast cell growth (Fig. 8A, B). These results suggest that *NAL1* involves in the regulation of cell division.

### NAL1is involved in crown root development

Besides its role in leaf development, *NAL1* was recently reported to have a novel function in promoting crown (or adventitious) root development [16]. To confirm this new finding in different genetic background, we examined the crown root phenotype of *nal1-2* and *nal1-3*. The crown root numbers are markedly reduced in *nal1-2* and *nal1-3*, compared with their wild type plant Nipponbare and F2-285, respectively (S6A-D Fig.). The rice *Crown Rootless* (*CRL*) genes are critical for crown root initiation and expressed in the shoot base region where crown roots differentiate [44–46]. RT-qPCR analysis showed that expression of *CRL1*, *CRL4*, and *CRL5* was significantly decreased in both *nal1-2* and *nal1-3* (S6E-F Fig.). Crown root initiation requires the initial cell division in the pericycle cells adjacent to the peripheral vascular cylinder in the stem [44]. *NAL1* may promote pericycle cell division during crown root formation via regulating expression of the *CRL* genes.

## Discussion

Our study revealed novel roles of *NAL1* during leaf growth and development, which was believed to regulate vein patterning and PAT [17]. We identified a new null allele of *NAL1* and designated it as *nal1-2*. Thickened leaves and internodes in *nal1-2* were the result of enhanced cell division in the periclinal orientation. Conversely, inhibition of cell division in the anticlinal direction was largely responsible for narrow, shortened leaves and half dwarf stature in *nal1-2*. However, *NAL1* did not affect the cell size and shape. We observed altered expression levels of cell cycle-regulating genes, which indicated that *NAL1* directly or indirectly regulates the expression of G1- and S-phase-specified genes. Heterogenous expression of *NAL*1 in fission yeast (*Schizosaccharomyces pombe*) inhibited cell division. Therefore, *NAL1* involves in cell cycle regulation.

Altered cell proliferation rates affect leaf shape and size, which has been reported in several previous studies. *SLENDER LEAF 1* (*SLE1*) was expressed specifically during the M-phase in the cell cycle. The *slender leaf 1* (*sle1*) mutants of rice showed impaired cell proliferation, resulting in narrow leaf blades [22]. In *Arabidopsis*, the prolonged S-phase duration inhibited leaf growth in *short-root* (*shr*) and *scarecrow* (*scr*) mutants [47]. These genes described above that affect the cell division rate were characterized by analyzing leaf epidermal cells, which show mostly anticlinal cell divisions. The orientation of the division plane is mainly periclinal (related to cell layers) and anticlinal (related to cell numbers). In this study, we showed that impaired anticlinal cell division accompanied an enhanced periclinal cell division in both leaves and internodes. These results suggest that the balance between anticlinal and periclinal cell division in leaves and internodes is disturbed in *nal1-2*, which reduces the size but increases the thickness of leaves, as well as shortens but thickens internodes. In plant cells, the plane of cell division is determined by the action of a cortical preprophase band (PPB), which forms during G2 and persists throughout prophase, but is disassembled upon the transition to metaphase. The disappearance of PPB leaves behind a cortical division site (CDS) to guide a new cell wall (cell plate) to form at the correct position [48]. NAL1 protein contained a putative nuclear localization signal, and its GFP–fusion protein was detected in the nucleus [17]. Thus, a direct or indirect relationship may exist between the NAL1 protein and the site of PPB formation. However, further studies are required to test this hypothesis.

We demonstrated that *NAL1* regulates the orientation of cell division and can occur as early as during leaf primordia initiation. In addition, the function of *NAL1* was not required for formation of the SAM. These results suggested that narrow leaves in *nal1-2* are correlated with the size of leaf primordia rather than the SAM. Leaf primordia initiated from a small mound of undifferentiated tissue at the tip of the stem. The number of leaf primordia founder cells recruited from the SAM was thought to correspond to the final leaf size [5]. For example, the *struwwelpeter* (*swp*) mutant in *Arabidopsis* showed altered expression of an RNA polymerase II transcription mediator and had reduced cell numbers in leaves, which was associated with very young leaf primordia containing decreased cell numbers. This decrease in cell number observed in very young primordia suggested that the numbers of initially recruited founder cells are reduced [49]. In rice, *nal1-2* suppressed the growth of young leaf primordia along the longitudinal axis, whereas the middle part of the leaf primordia thickened at an enhanced rate. Compared with the WT, we observed no difference in the shape and size of the SAM in *nal1-2*. Thus, the mechanism controlling cell number during leaf primordium initiation may be associated with the final leaf shape and size, which is not affected by the shape and size of the SAM.

The downregulated transcript level of *PIN1* was consistent with previous studies reporting that *NAL1* participates in the regulation of PAT [17]. Our results showed that *NAL1* also plays a role in regulating the expression of *AUXIN RESPONSE FACTOR*s (*ARF*s) and is not involved in tryptophan-dependent auxin biosynthesis. In maize, the application of the PAT inhibitor N-1-naphthylphthalamic acid disrupts leaf initiation and formation of leaf margins [50]. The auxin response factors (ARFs) are required for controlling the expression of auxin response genes, which are involved in auxin signaling [41]. These results suggest that *NAL1* may affect both PAT and auxin signaling. Previous studies have shown that *YABBY* gene expression is required for leaf development. In rice, the overexpression of *WUSCHEL-LIKE HOMEOBOX3* (*WOX3*) (identical to *NAL2/3*) repressed expression of *YABBY3*, resulting in twisted and knotted leaves [51]. In the mutant *nal2/3*, expression of the *YABBY* family was altered [52]. Mutants of *drooping leaf* (*dl*), a member of the *YABBY* family, were defective in midrib formation in leaves. Ectopic expression of *DL* resulted in ectopically formed clear cells, as well as abnormal adaxial vascular bundles [43, 53]. In the *nal1-2* mutant, the expression levels of *YABBY1*, *YABBY2*, and *YABBY6* were reduced by almost 50% compared to the WT. Moreover, the midrib structure in *nal1-2* leaves was abnormal, the features of which include shrinked clear cells and increased adaxial vascular bundle size. Based on these results, the NAL1 protein may function with the YABBY family (directly and/or indirectly) to regulate the formation of normal midribs in leaves.

## Supporting Information

S1 Fig Leaf epidermal cell size and outer and inner diameters of internodes. (A, B) Comparison of epidermal cell width and length at the middle part of the upper second leaves between WT and *nal1-2* plants. (C, D) Comparison of the outer and inner diameter of the upper four internodes between wild-type (WT) and *nal1-2* plants. Each column represents the mean ± standard error (SE) (n ≥ 10). Asterisks show significant differences based on Student’s *t*-test: *, 0.01 ≤ *P* < 0.01; **, *P* < 0.01.
(TIF)

S2 Fig Results of the genome-walking trial. Four DNA fragments were obtained from three AP primers. Red arrows indicate DNA fragments.
(TIF)

S3 Fig Results of polymerase chain reaction (PCR) using primers 4-1. The length of the predicted DNA fragment in the *nal1-2* mutant is 912 bp, whereas the predicted DNA fragment in the wild type (WT) is too large (11,532 bp) to be amplified using a single PCR. We obtained the predicted DNA fragment size in *nal1-2*.
(TIF)

S4 Fig The trypsin-like serine and cysteine protease domains of the NAL1 protein are highly conserved. Alignment was performed using the deduced amino acid sequences of NAL1 with homologous proteins. Red box indicates the trypsin-like serine and cysteine protease domains.
(TIF)

S5 Fig Phenotypes of *nal1-3*. (A) Morphology of wild type (F2-285A, left) and *nal1-3* (right) plants at the heading stage (bar = 5 cm). (B) Comparison of leaf width between the WT (left) and *nal1-3* (right) (bar = 5 mm). (C) Comparison of leaf length between the WT (left) and *nal1-3* (right) (bar = 5 cm). (D, E) Comparison of transverse sections of internode III between the WT (left) and *nal1-3* (right). At the upper left corner, images of transverse sections of internode are zoomed-in (bars = 50 μm). (F) Transverse sections through the middle part of the mature leaves of WT (lower) and *nal1-3* plants (upper) (bar = 100 μm).
(TIF)

S6 Fig Crown root phenotype of *nal1-2* and *nal1-3*. (A) Crown root morphology of one-week-old seedlings of wild-type plant (left: Nipponbare) and *nal1-2* mutant (right). (B) Crown root morphology of one-week-old seedlings of wild-type plant (left: F2-285) and *nal1-3* mutant (right). (C, D) The numbers of crown root in one-week-old seedlings of wild-type plant, *nal1-2* and *nal1-3*. Data are shown as means ± standard error (SE) (n ≥ 10). Student’s t-test was used to analyze significant differences between the WT and mutants. **, P < 0.01. (E, F) Relative expression level of genes involved in crown root development in shoot base of one-week-old seedlings of wild-type, *nal1-2* (E) and *nal1-3* (F) mutant. Each gene was normalized to *Ubiquitin*. The RT-qPCR analysis was replicated for three times with similar result. Student’s t-test was used to analyze significant differences between the WT and mutants. **, P < 0.01.
(TIF)

S1 Table Primers used for genetic mapping, qRT-PCR, semi-quantitative RT-PCR analyses and genome walking trials. (DOC)

S2 Table Morphometric analysis of the *nal1-3* mutant. Phenotypes of *nal1-3* were measured at the early heading stage. Second leaf is the upper second leaf. Values are the mean ± standard error (SE) (n≥15). Asterisks reveal the significance of differences between wild-type and *nal1-3* plants, which is obtained by Student’s *t*-test: **, *P* < 0.01.
(DOC)

## References

[1] Lyndon RF, Cunninghame ME (1986) Control of shoot apical development via cell division. Symp Soc Exp Biol 40: 233–255. 3544304

[2] Reinhardt D, Mandel T, Kuhlemeier C (2000) Auxin regulates the initiation and radial position of plant lateral organs. Plant Cell 12: 507–518. 10760240 10.1105/tpc.12.4.507 PMC139849

[3] Fleming A, McQueen-Mason S, Mandel T, Kuhlemeier C (1997) Induction of leaf primordia by the cell wall protein expansion. Science 276: 1415–1418.

[4] Bowman JL, Eshed Y, Baum SF (2002) Establishment of polarity in angiosperm lateral organs. Trends Genet 18: 134–141. 11858837 10.1016/s0168-9525(01)02601-4

[5] Gonzalez N, Vanhaeren H, Inze D (2012) Leaf size control: complex coordination of cell division and expansion. Trends Plant Sci 17: 332–340. 10.1016/j.tplants.2012.02.003 22401845

[6] Narita NN, Moore S, Horiguchi G, Kubo M, Demura T, et al (2004) Overexpression of a novel small peptide ROTUNDIFOLIA4 decreases cell proliferation and alters leaf shape in Arabidopsis thaliana. Plant J 38: 699–713. 15125775 10.1111/j.1365-313X.2004.02078.x

[7] Lee BH, Ko JH, Lee S, Lee Y, Pak JH, et al (2009) The Arabidopsis GRF-INTERACTING FACTOR gene family performs an overlapping function in determining organ size as well as multiple developmental properties. Plant Physiol 151: 655–668. 10.1104/pp.109.141838 19648231 PMC2754652

[8] Eloy NB, de Freitas Lima M, Van Damme D, Vanhaeren H, Gonzalez N, et al (2011) The APC/C subunit 10 plays an essential role in cell proliferation during leaf development. Plant J 68: 351–363. 10.1111/j.1365-313X.2011.04691.x 21711400

[9] Kim JH, Choi D, Kende H (2003) The AtGRF family of putative transcription factors is involved in leaf and cotyledon growth in Arabidopsis. Plant J 36: 94–104. 12974814 10.1046/j.1365-313x.2003.01862.x

[10] Smith LG (2001) Plant cell division: building walls in the right places. Nat Rev Mol Cell Biol 2: 33–39. 11413463 10.1038/35048050

[11] Errera L (1888) Über Zellformen und Siefenblasen. Botanisches Centralblatt 34: 395–399.

[12] Lewis D, Bacic A, Chandler PM, Newbigin EJ (2009) Aberrant cell expansion in the elongation mutants of barley. Plant Cell Physiol 50: 554–571. 10.1093/pcp/pcp015 19181700

[13] Chandler PM, Robertson M (1999) Gibberellin dose-response curves and the characterization of dwarf mutants of barley. Plant Physiol 120: 623–632. 10364415 10.1104/pp.120.2.623 PMC59302

[14] Kessler S, Seiki S, Sinha N (2002) Xcl1 causes delayed oblique periclinal cell divisions in developing maize leaves, leading to cellular differentiation by lineage instead of position. Development 129: 1859–1869. 11934852 10.1242/dev.129.8.1859

[15] De Rybel B, Moller B, Yoshida S, Grabowicz I, Barbier de Reuille P, et al (2013) A bHLH complex controls embryonic vascular tissue establishment and indeterminate growth in Arabidopsis. Dev Cell 24: 426–437. 10.1016/j.devcel.2012.12.013 23415953

[16] Cho S, Yoo S, Zhang H, Lim J, Paek N (2014) Rice NARROW LEAF1 regulates leaf and adventitious root development. Plant Molecular Biology Reporter 32: 270–281.

[17] Qi J, Qian Q, Bu Q, Li S, Chen Q, et al (2008) Mutation of the rice Narrow leaf1 gene, which encodes a novel protein, affects vein patterning and polar auxin transport. Plant Physiol 147: 1947–1959. 10.1104/pp.108.118778 18562767 PMC2492643

[18] Chen M, Luo J, Shao G, Wei X, Tang S, et al (2012) Fine mapping of a major QTL for flag leaf width in rice, qFLW4, which might be caused by alternative splicing of *NAL1* . Plant Cell Rep 31: 863–872. 10.1007/s00299-011-1207-7 22179305

[19] Takai T, Adachi S, Taguchi-Shiobara F, Sanoh-Arai Y, Iwasawa N, et al (2013) A natural variant of *NAL1*, selected in high-yield rice breeding programs, pleiotropically increases photosynthesis rate. Sci Rep 3: 2149 10.1038/srep02149 23985993 PMC3756344

[20] Fujita D, Trijatmiko KR, Tagle AG, Sapasap MV, Koide Y, et al (2013) *NAL1* allele from a rice landrace greatly increases yield in modern indica cultivars. Proc Natl Acad Sci U S A 110: 20431–20436. 10.1073/pnas.1310790110 24297875 PMC3870739

[21] Zhang GH, Li SY, Wang L, Ye WJ, Zeng DL, et al (2014) LSCHL4 from Japonica Cultivar, which is allelic to *NAL1*, increases yield of indica super rice 93-11. Mol Plant 7: 1350–1364. 10.1093/mp/ssu055 24795339 PMC4115278

[22] Yoshikawa T, Eiguchi M, Hibara K, Ito J, Nagato Y (2013) Rice slender leaf 1 gene encodes cellulose synthase-like D4 and is specifically expressed in M-phase cells to regulate cell proliferation. J Exp Bot 64: 2049–2061. 10.1093/jxb/ert060 23519729 PMC3638827

[23] Maundrell K (1990) nmt1 of fission yeast. A highly transcribed gene completely repressed by thiamine. J Biol Chem 265: 10857–10864. 2358444

[24] Cebolla A, Vinardell JM, Kiss E, Olah B, Roudier F, et al (1999) The mitotic inhibitor ccs52 is required for endoreduplication and ploidy-dependent cell enlargement in plants. EMBO J 18: 4476–4484. 10449413 10.1093/emboj/18.16.4476 PMC1171522

[25] Takada S, Takada N and Yoshida A (2013) ATML1 promotes epidermal cell differentiation in Arabidopsis shoots. Development 140: 1919–1923. 10.1242/dev.094417 23515472

[26] Howell S (1998) Molecular Genetics of Plant Development Cambridge University Press, Cambridge, UK

[27] Lyndon R (1998) The Shoot Apical Meristem: Its Growth and Development Cambridge University Press, Cambridge, UK

[28] De Veylder L, Beeckman T, Beemster GT, Krols L, Terras F, et al (2001) Functional analysis of cyclin-dependent kinase inhibitors of Arabidopsis. Plant Cell 13: 1653–1668. 11449057 10.1105/TPC.010087 PMC139548

[29] Nelson T and Dengler N (1997) Leaf Vascular Pattern Formation. Plant Cell 9: 1121–1135. 12237378 10.1105/tpc.9.7.1121 PMC156985

[30] Scarpella E, Rueb S, Meijer AH (2003) The RADICLELESS1 gene is required for vascular pattern formation in rice. Development 130: 645–658. 12505996 10.1242/dev.00243

[31] Fabian-Marwedel T, Umeda M, Sauter M (2002) The rice cyclin-dependent kinase-activating kinase R2 regulates S-phase progression. Plant Cell 14: 197–210. 11826308 10.1105/tpc.010386 PMC150560

[32] Zhao SQ, Hu J, Guo LB, Qian Q, Xue HW (2010) Rice leaf inclination2, a VIN3-like protein, regulates leaf angle through modulating cell division of the collar. Cell Res 20: 935–947. 10.1038/cr.2010.109 20644566

[33] Renaudin JP, Doonan JH, Freeman D, Hashimoto J, Hirt H, et al (1996) Plant cyclins: a unified nomenclature for plant A-, B- and D-type cyclins based on sequence organization. Plant Mol Biol 32: 1003–1018. 9002599 10.1007/BF00041384

[34] Sauter M (1997) Differential expression of a CAK (cdc2-activating kinase)-like protein kinase, cyclins and cdc2 genes from rice during the cell cycle and in response to gibberellin. Plant J 11: 181–190. 9076986 10.1046/j.1365-313x.1997.11020181.x

[35] Colon-Carmona A, You R, Haimovitch-Gal T, Doerner P (1999) Technical advance: spatio-temporal analysis of mitotic activity with a labile cyclin-GUS fusion protein. Plant J 20: 503–508. 10607302 10.1046/j.1365-313x.1999.00620.x

[36] Genschik P, Criqui MC, Parmentier Y, Derevier A, Fleck J (1998) Cell cycle-dependent proteolysis in plants. Identification Of the destruction box pathway and metaphase arrest produced by the proteasome inhibitor mg132. Plant Cell 10: 2063–2076. 9836745 10.1105/tpc.10.12.2063 PMC143975

[37] Guo J, Wang F, Song J, Sun W, Zhang XS (2010) The expression of Orysa;CycB1;1 is essential for endosperm formation and causes embryo enlargement in rice. Planta 231: 293–303. 10.1007/s00425-009-1051-y 19921249

[38] Galweiler L, Guan C, Muller A, Wisman E, Mendgen K, et al (1998) Regulation of polar auxin transport by AtPIN1 in Arabidopsis vascular tissue. Science 282: 2226–2230. 9856939 10.1126/science.282.5397.2226

[39] Friml J, Wisniewska J, Benkova E, Mendgen K, Palme K (2002) Lateral relocation of auxin efflux regulator PIN3 mediates tropism in Arabidopsis. Nature 415: 806–809. 11845211 10.1038/415806a

[40] Zhao Y, Christensen SK, Fankhauser C, Cashman JR, Cohen JD, et al (2001) A role for flavin monooxygenase-like enzymes in auxin biosynthesis. Science 291: 306–309. 11209081 10.1126/science.291.5502.306

[41] Guilfoyle TJ and Hagen G (2007) Auxin response factors. Curr Opin Plant Biol 10: 453–460. 17900969 10.1016/j.pbi.2007.08.014

[42] Bowman JL (2000) The YABBY gene family and abaxial cell fate. Curr Opin Plant Biol 3: 17–22. 10679447 10.1016/s1369-5266(99)00035-7

[43] Yamaguchi T, Nagasawa N, Kawasaki S, Matsuoka M, Nagato Y, et al (2004) The YABBY gene DROOPING LEAF regulates carpel specification and midrib development in Oryza sativa. Plant Cell 16: 500–509. 14729915 10.1105/tpc.018044 PMC341919

[44] Liu H, Wang S, Yu X, Yu J, He X, et al (2005) ARL1, a LOB-domain protein required for adventitious root formation in rice. Plant J 43:47–56. 15960615 10.1111/j.1365-313X.2005.02434.x

[45] Liu S, Wang J, Wang L, Wang X, Xue Y, et al (2009) Adventitious root formation in rice requires OsGNOM1 and is mediated by the OsPINs family. Cell Res 19:1110–1119. 10.1038/cr.2009.70 19546891

[46] Kitomi Y, Ito H, Hobo T, Aya K, Kitano H, et al (2011) The auxin responsive AP2/ERF transcription factor CROWN ROOTLESS5 is involved in crown root initiation in rice through the induction of OsRR1, a type-A response regulator of cytokinin signaling. Plant J 67:472–484. 10.1111/j.1365-313X.2011.04610.x 21481033

[47] Dhondt S, Coppens F, De Winter F, Swarup K, Merks RM, et al (2010) SHORT-ROOT and SCARECROW regulate leaf growth in Arabidopsis by stimulating S-phase progression of the cell cycle. Plant Physiol 154: 1183–1195. 10.1104/pp.110.158857 20739610 PMC2971598

[48] Rasmussen CG, Humphries JA, Smith LG (2011) Determination of symmetric and asymmetric division planes in plant cells. Annu Rev Plant Biol 62: 387–409. 10.1146/annurev-arplant-042110-103802 21391814

[49] Autran D, Jonak C, Belcram K, Beemster GT, Kronenberger J, et al (2002) Cell numbers and leaf development in Arabidopsis: a functional analysis of the STRUWWELPETER gene. EMBO J 21: 6036–6049. 12426376 10.1093/emboj/cdf614 PMC137206

[50] Scanlon MJ (2003) The polar auxin transport inhibitor N-1-naphthylphthalamic acid disrupts leaf initiation, KNOX protein regulation, and formation of leaf margins in maize. Plant Physiol 133: 597–605. 14500790 10.1104/pp.103.026880 PMC219036

[51] Dai M, Hu Y, Zhao Y, Liu H, Zhou DX (2007) A WUSCHEL-LIKE HOMEOBOX gene represses a YABBY gene expression required for rice leaf development. Plant Physiol 144: 380–390. 17351053 10.1104/pp.107.095737 PMC1913789

[52] Cho SH, Yoo SC, Zhang H, Pandeya D, Koh HJ, et al (2013) The rice narrow leaf2 and narrow leaf3 loci encode WUSCHEL-related homeobox 3A (OsWOX3A) and function in leaf, spikelet, tiller and lateral root development. New Phytol 198: 1071–1084. 10.1111/nph.12231 23551229

[53] Nagasawa N, Miyoshi M, Sano Y, Satoh H, Hirano H, et al (2003) SUPERWOMAN1 and DROOPING LEAF genes control floral organ identity in rice. Development 130: 705–718. 12506001 10.1242/dev.00294

