# Supplementary figures and images for: Characterization of a Null Allelic Mutant of the Rice NAL1 Gene Reveals Its Role in Regulating Cell Division
Source: PLoS One. 2015 Feb 6;10(2):e0118169. doi: 10.1371/journal.pone.0118169 (PMC4320051; doi:10.1371/journal.pone.0118169)

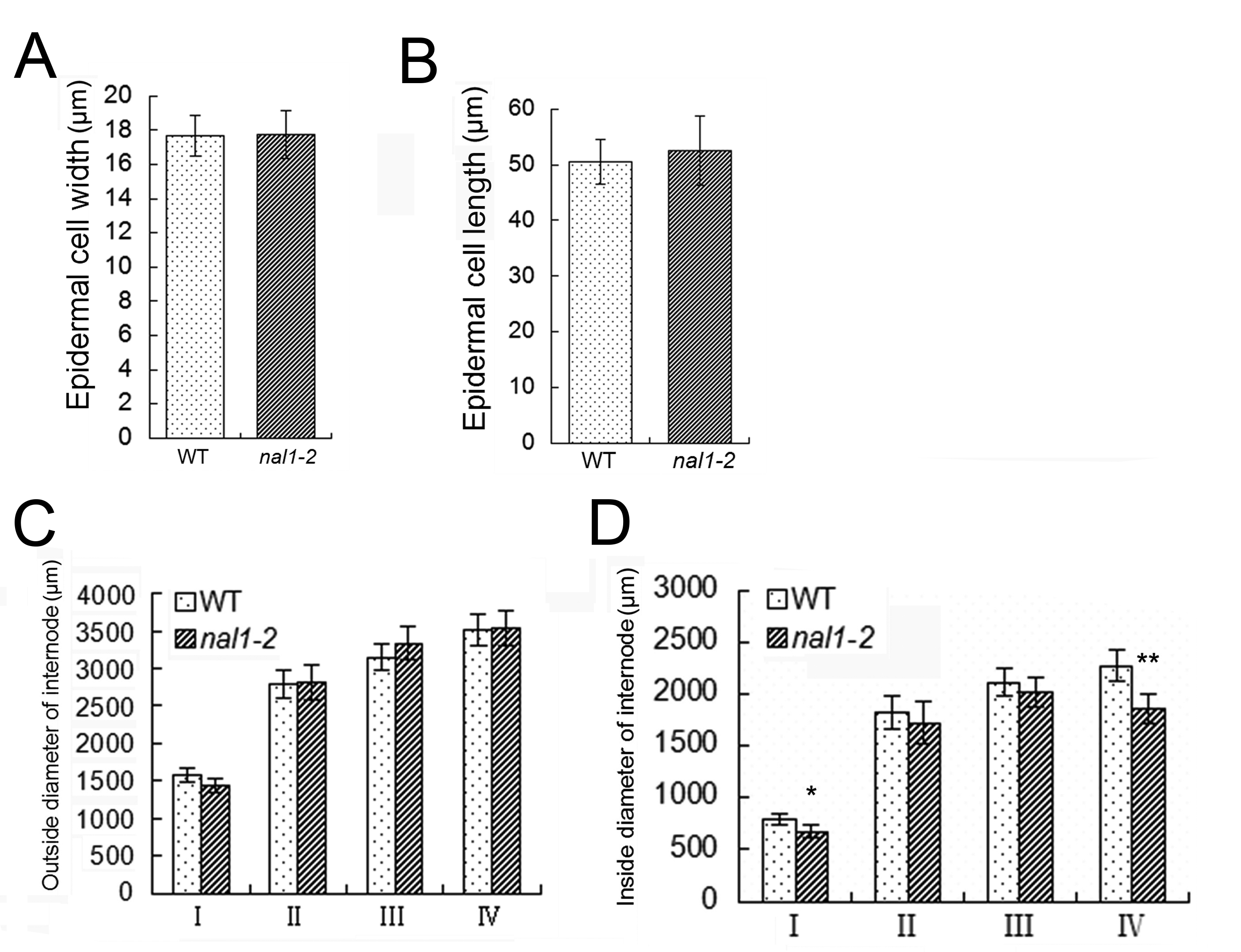

Supplement: S1 Fig — (A, B) Comparison of epidermal cell width and length at the middle part of the upper second leaves between WT and nal1-2 plants. (C, D) Comparison of the outer and inner diameter of the upper four internodes between wild-type (WT) and nal1-2 plants. Each column represents the mean ± standard error (SE) (n ≥ 10). Asterisks show significant differences based on Student’s t-test: *, 0.01 ≤ P < 0.01; **, P < 0.01. (TIF) [file pone.0118169.s001.tif]

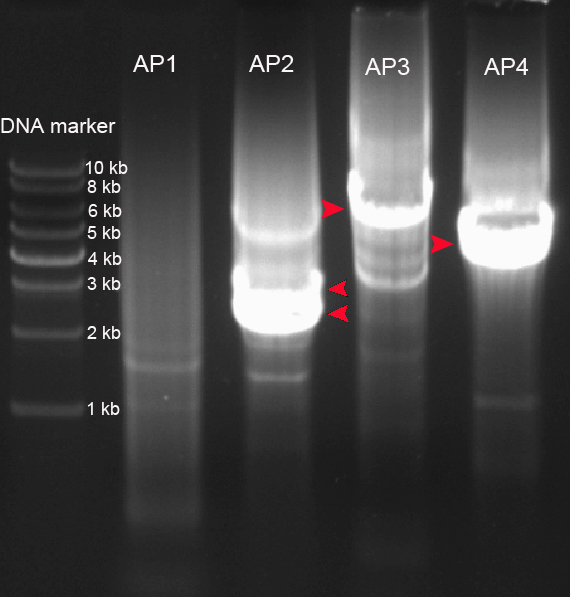

Supplement: S2 Fig — Four DNA fragments were obtained from three AP primers. Red arrows indicate DNA fragments. (TIF) [file pone.0118169.s002.tif]

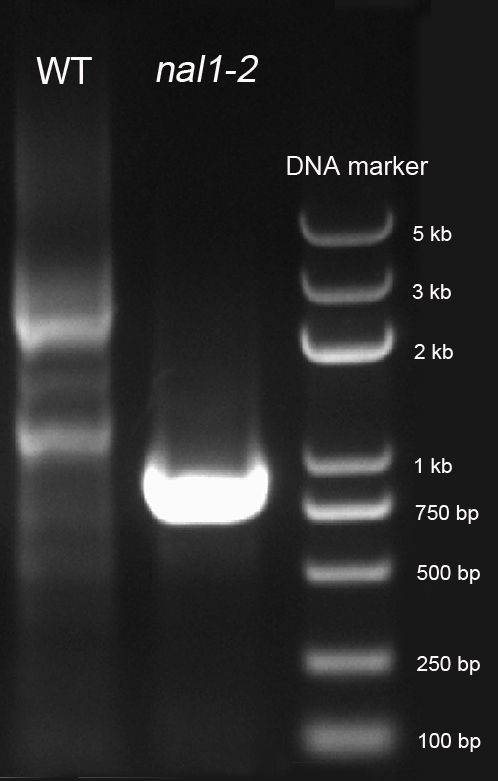

Supplement: S3 Fig — The length of the predicted DNA fragment in the nal1-2 mutant is 912 bp, whereas the predicted DNA fragment in the wild type (WT) is too large (11,532 bp) to be amplified using a single PCR. We obtained the predicted DNA fragment size in nal1-2. (TIF) [file pone.0118169.s003.tif]

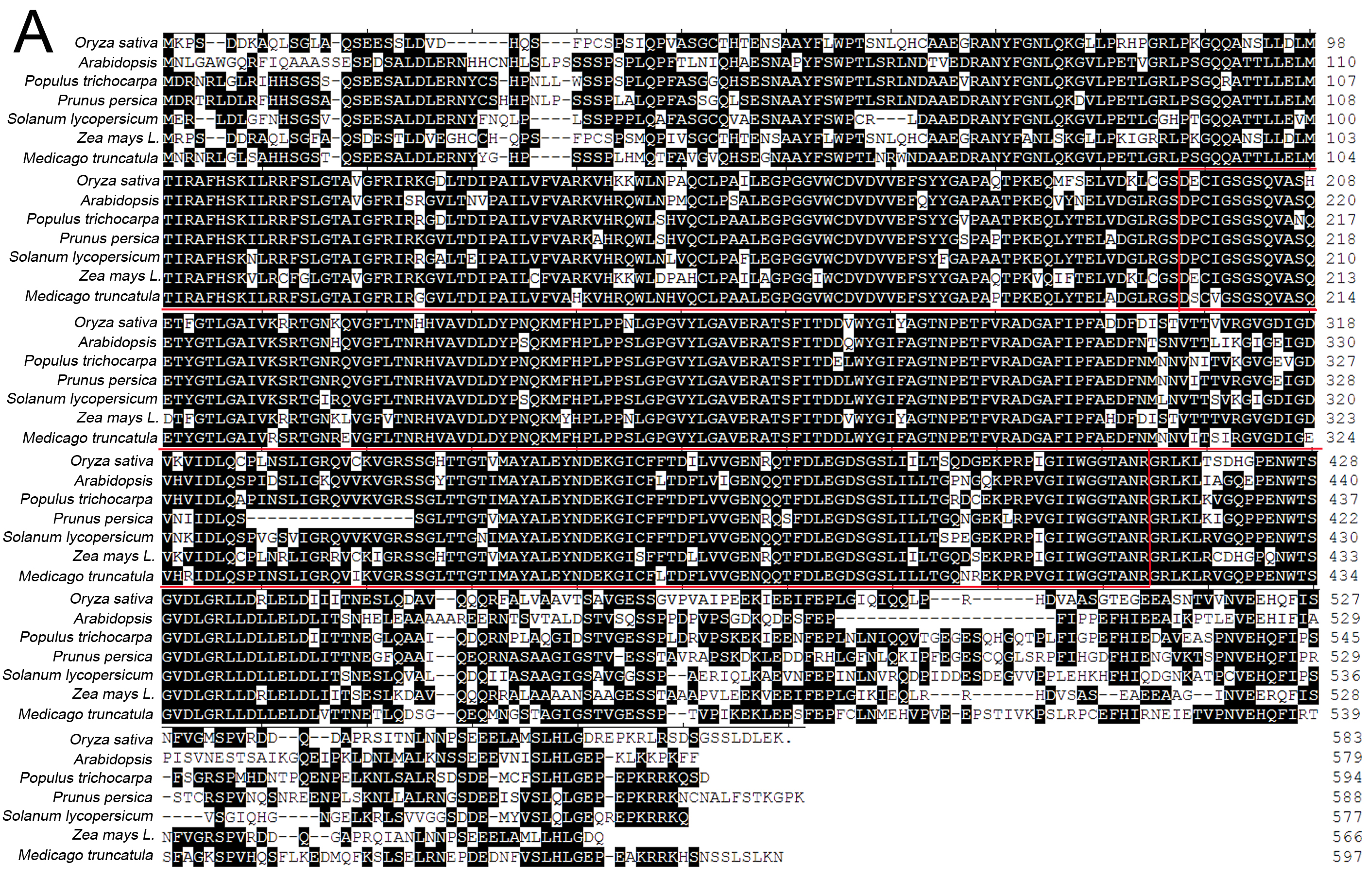

Supplement: S4 Fig — Alignment was performed using the deduced amino acid sequences of NAL1 with homologous proteins. Red box indicates the trypsin-like serine and cysteine protease domains. (TIF) [file pone.0118169.s004.tif]

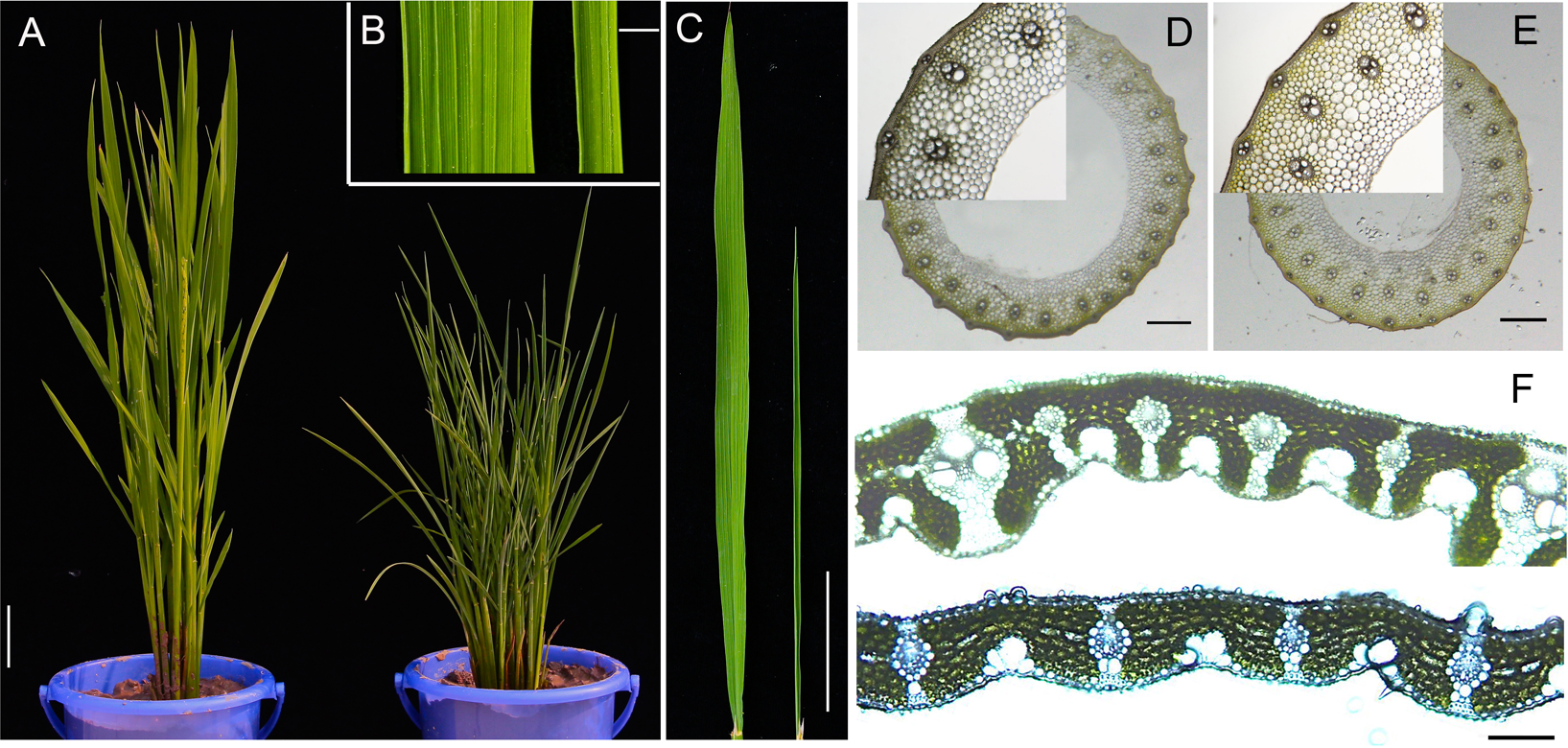

Supplement: S5 Fig — (A) Morphology of wild type (F2-285A, left) and nal1-3 (right) plants at the heading stage (bar = 5 cm). (B) Comparison of leaf width between the WT (left) and nal1-3 (right) (bar = 5 mm). (C) Comparison of leaf length between the WT (left) and nal1-3 (right) (bar = 5 cm). (D, E) Comparison of transverse sections of internode III between the WT (left) and nal1-3 (right). At the upper left corner, images of transverse sections of internode are zoomed-in (bars = 50 μm). (F) Transverse sections through the middle part of the mature leaves of WT (lower) and nal1-3 plants (upper) (bar = 100 μm). (TIF) [file pone.0118169.s005.tif]

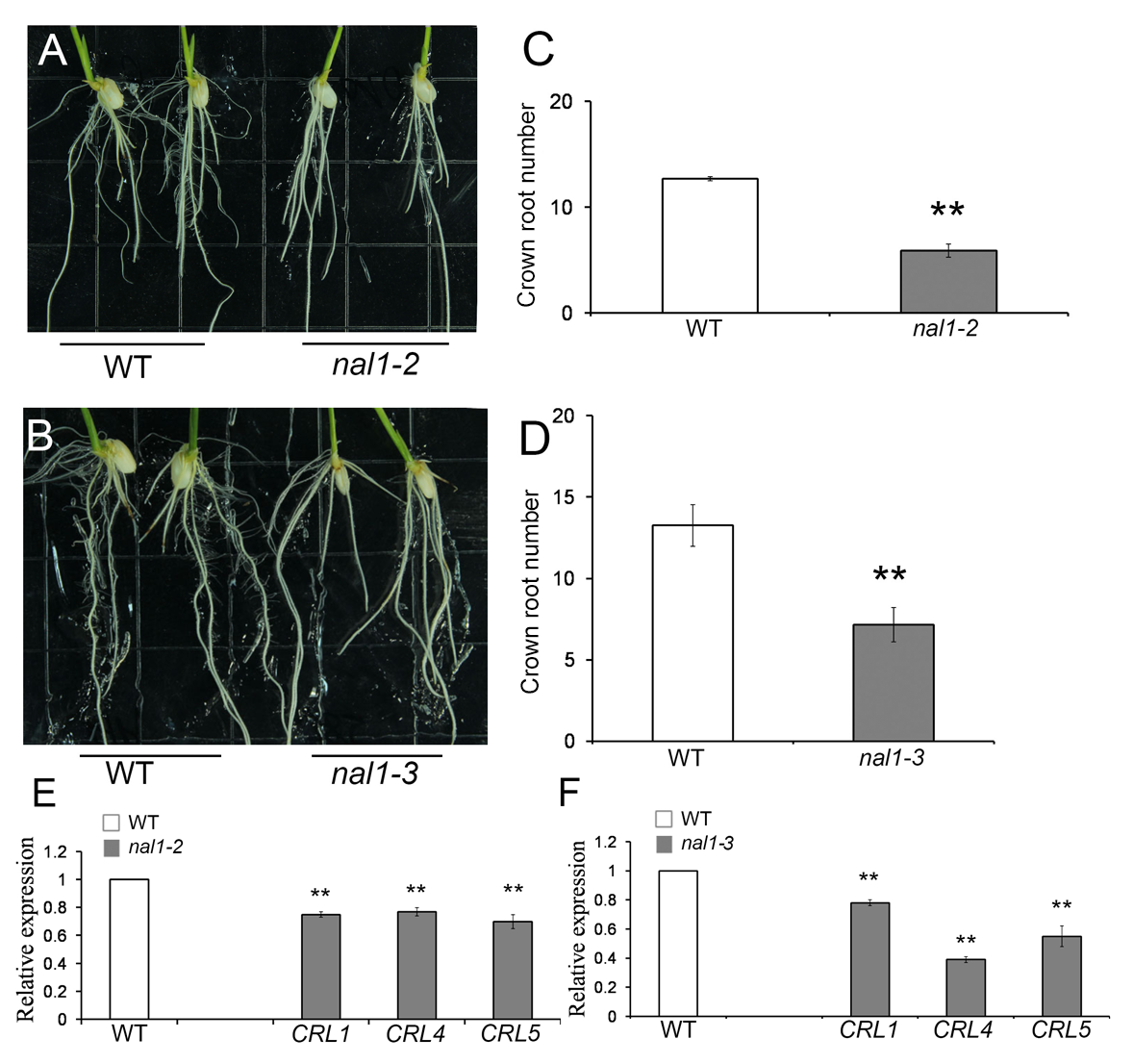

Supplement: S6 Fig — (A) Crown root morphology of one-week-old seedlings of wild-type plant (left: Nipponbare) and nal1-2 mutant (right). (B) Crown root morphology of one-week-old seedlings of wild-type plant (left: F2-285) and nal1-3 mutant (right). (C, D) The numbers of crown root in one-week-old seedlings of wild-type plant, nal1-2 and nal1-3. Data are shown as means ± standard error (SE) (n ≥ 10). Student’s t-test was used to analyze significant differences between the WT and mutants. **, P < 0.01. (E, F) Relative expression level of genes involved in crown root development in shoot base of one-week-old seedlings of wild-type, nal1-2 (E) and nal1-3 (F) mutant. Each gene was normalized to Ubiquitin. The RT-qPCR analysis was replicated for three times with similar result. Student’s t-test was used to analyze significant differences between the WT and mutants. **, P < 0.01. (TIF) [file pone.0118169.s006.tif]
