# Supplementary material for: Characterization of a Null Allelic Mutant of the Rice NAL1 Gene Reveals Its Role in Regulating Cell Division
Source: PLoS One. 2015 Feb 6;10(2):e0118169. doi: 10.1371/journal.pone.0118169 (PMC4320051; doi:10.1371/journal.pone.0118169)
Supplement: S1 Table — (DOC) [file pone.0118169.s007.doc]

**Table.S1.** Primers used for genetic mapping, qRT-PCR, semi-quantitative RT-PCR analyses and genome walking trials.

| Primer Name | Primer Sequence | Restriction enzyme |
| --- | --- | --- |
| M4-1 | F, 5’-CTCCCATAACCAAAAGCTCC-3’  R, 5’-ATGTTGGCTCTCTAAACCCG-3’ |  |
| M4-2 | F, 5’-GGATGACTGATCCTTGTCTTC-3’  R, 5’-GATCGTGATTCCATGATGCTG-3’ |  |
| M4-3 | F, 5’-ATCGATCCATCAGACGCCTA-3’  R, 5’-TGAGCGGATTAGCAGCACGTA-3’ |  |
| M4-4 | F, 5’-CAGGAGAGATATTACACAGG-3’  R, 5’-CCGATGATATCTGCAAGCCA-3’ |  |
| M4-5 | F, 5’-CCAAGAGCTACTAGGTACCT-3’  R, 5’-GGCTCATTATTAGTGGTGGC-3’ |  |
| C4-1 | F, 5’-CAGCCCATACTACTCCTTTC-3’  R, 5’-AGCTACTGGCAGATGACCAA-3’ | AlfⅢ |
| C4-2 | F, 5’-ATCGAAGAGCTCCTTCTGCCT-3’  R, 5’-CCGTGTGTTGACTGCTAACT-3’ | AciⅠ |
| C4-3 | F, 5’-CGTACATTGTGAAATGGAGGG-3’  R, 5’-TTCACCAGCATGCAAGACCA-3’ | PsiⅠ |
| C4-4 | F, 5’-ACTAGTTCCAGAACCATCCG-3’  R, 5’-CCTCACACGCGGTTTTATCA-3’ | AciⅠ |
| Primer1-1 | F, 5’-GCATTCGTTATCTACCTGTC-3’  R, 5’-CTCTGAAGAACCAGAATCAG-3’ |  |
| primer2-1 | F, 5’-CTTGATGTGGATCACCAGTC-3’  R, 5’-CCAATACATTCGTCACTGCC-3’ |  |
| primer3-1 | F, 5’-GAGGTGTTTGGTGTGATGTTG-3’  R, 5’-CGAAGTTTGAGATGAACTGG-3’ |  |
| Primer4-1 | F, 5’-GCTATTGGCTGATTGTCAAGG-3’  R, 5’-CTGTCCTAGAGCATCTCAAC-3’ |  |
| SP1 | 5’-GAGCAGAGCATCCCAACAAACTG-3’ |  |
| SP2 | 5’-CTACACACCAAACACCTCCTGGAC-3’ |  |
| SP3 | 5’-CCACTTCTTATGAACCTTGCGAGC-3’ |  |
| CDKA;1 | F, 5’-ATCACGGCAACATCGTCAGG-3’  R, 5’-AGTAAGCAACGCCGCGGAGTA-3’ |  |
| CDKA;2 | F, 5’-ACCACCGCATAGTCAAATCGTT-3’  R, 5’-ACCACAATGTCACCACCTCGTGA-3’ |  |
| R2 | F, 5’-TCTGCACCTCCACTTCGCTCA-3’  R, 5’-TAGGTGGTGGCCTTGGAAGCT-3’ |  |
| H3 | F, 5’-GCAAGTACCAGAAGAGCACG-3’  R, 5’-GTTGGTGTCCTCGAAGAGAC-3’ |  |
| H4 | F, 5’-GCAAGTACCAGAAGAGCACG-3’  R, 5’-AGAGGTTGGTGTCCTCGAAG-3’ |  |
| CYCB1;1 | F, 5’-GCTTCAGTGGAATCTCACCG-3’  R, 5’-GTGGTGCTTGAGAGTGTCAG-3’ |  |
| PIN1 | F, 5’-AGTCAAGGGGAGGACAGGAT-3’  R, 5’-TCCGTTTTACAAGGGTCAGC-3’ |  |
| PIN3 | F, 5’-CTCTACCACAAGGGATTGTG-3’  R, 5’-CATACGCTGTTTGAGATGGAC-3’ |  |
| ARF1 | F, 5’-CCAAGAGATCCAAAGCCATCAG-3’  R, 5’-CCAAGGATCATCTCCAACTTC-3’ |  |
| ARF2 | F, 5’-GTCCGTTCTGGCTCTCAATC-3’  R, 5’-GGGAAGGCTGTGGAGATACA-3’ |  |
| ARF3 | F, 5’-TCTCGTCCCTCTACGTGCTT-3’  R, 5’-CCGATTACAACGGGAGCTTA-3’ |  |
| YUCCA1 | F, 5’-ACCTCATCCTCGGTAACACG-3’  R, 5’-TCTCCTTCACTGCTCCCACT-3’ |  |
| YUCCA2 | F, 5’-GAAGACTGTCCTTGTTGTAG-3’  R, 5’-GATGTATCACCAAGAATCGC-3’ |  |
| YABBY1 | F, 5’-GTCGGTCCAGTTTACATCGG-3’  R, 5’-CCTCTGTTCTGTACCTGGTG-3’ |  |
| YABBY2 | F, 5’-CACTGCAACTTCTGCAACAC-3’  R, 5’-GCCATATCTGGAAGATGAGCC-3’ |  |
| YABBY6 | F, 5’-CTTCTGCAACACGATTCTCG-3’  R, 5’-CACTTTGATCACGGAAGCATC-3’ |  |
| Ubiquitin | F, 5’-GTCTGATCTTCGCTGGCAAGCAGC-3’  R, 5’-GCATACTGCTGTCCCACAGGAAACTG-3’ |  |
| CRL1 | F, 5’- CATCTATGGCTGCGTCGCCC-3’  R, 5’- CGCCTTGAGCGACGCCAGCT-3’ |  |
| CRL4 | F, 5’- TGTGCCTTCAGAGCTCCACA-3’  R, 5’- CTGCGAGTGATTCTGGCTGA-3’ |  |
| CRL5 | F, 5’- ATCGGCCGAGTCTCCGGCAA-3’  R, 5’- TTGATCGCCGCCACGTCGTA-3’ |  |
