# Supplementary material for: Characterization of a Null Allelic Mutant of the Rice NAL1 Gene Reveals Its Role in Regulating Cell Division
Source: PLoS One. 2015 Feb 6;10(2):e0118169. doi: 10.1371/journal.pone.0118169 (PMC4320051; doi:10.1371/journal.pone.0118169)
Supplement: S2 Table — Phenotypes of nal1-3 were measured at the early heading stage. Second leaf is the upper second leaf. Values are the mean ± standard error (SE) (n≥15). Asterisks reveal the significance of differences between wild-type and nal1-3 plants, which is obtained by Student’s t-test: **, P < 0.01. (DOC) [file pone.0118169.s008.doc]

| Traits | Wild Type | *nal1-3* |
| --- | --- | --- |
| Plant height (cm) | 67.59±3.14 | 44.68±2.41** |
| Blade length of flag leaf (cm) | 22.56±2.23 | 21.04±2.14** |
| Blade length of second leaf (cm) | 31.17±2.18 | 28.59±1.80* |
| Blade width of flag leaf (mm) | 16.27±0.47 | 5.09±0.46** |
| Blade width of second leaf (mm) | 11.52±0.69 | 5.20±0.33** |
| Tiller number | 9.63±1.32 | 21.75±5.49** |

**Table S2.** Morphometric analysis of the *nal1-3* mutant. Phenotypes of *nal1-3* were measured at the early heading stage. Second leaf is the upper second leaf. Values are the mean ± standard error (SE) (n≥15). Asterisks reveal the significance of differences between wild-type and *nal1-3* plants, which is obtained by Student’s *t*-test: **, *P* < 0.01.
